# Thioxanthone-Based Siloxane Photosensitizer for Cationic/Radical Photopolymerization and Photoinduced Sol–Gel Reactions

**DOI:** 10.3390/molecules29010255

**Published:** 2024-01-03

**Authors:** Thi-Thanh-Tam Nguyen, Louise Breloy, Agustin Rios De Anda, Hassan Hayek, Annalisa Chiappone, Jean-Pierre Malval, Daniel Grande, Davy-Louis Versace

**Affiliations:** 1 University Paris-Est Creteil, CNRS, ICMPE, UMR 7182, 94320 Thiais, France; thi-thanh-tam.nguyen@u-pec.fr (T.-T.-T.N.); agustin.rios-de-anda@u-pec.fr (A.R.D.A.);; 2 Dipartimento di Scienze Chimiche e Geologiche, Università degli Studi di Cagliari, Via Università 40, 09124 Cagliari, Italy; 3 Institut de Science des Matériaux de Mulhouse, UMR CNRS 7361, Université de Haute Alsace, 15 Rue Jean Starcky, 68057 Mulhouse, France

**Keywords:** photosensitizer, visible-light irradiation, free-radical photopolymerization, cationic photopolymerization, thioxanthone, photoinduced sol–gel reactions

## Abstract

In this investigation, a multifunctional visible-light TX-based photosensitizer containing a siloxane moiety (TXS) was designed with a good overall yield of 54%. The addition of a siloxane moiety enabled the incorporation of a TX photosensitizer into a siloxane network by photoinduced sol–gel chemistry, thus avoiding its release. Both liquid ^1^H and solid-state ^29^Si NMR measurements undeniably confirmed the formation of photoacids resulting from the photolysis of the TXS/electron acceptor molecule (Iodonium salt), which promoted the photoinduced hydrolysis/condensation of the trimethoxysilane groups of TXS, with a high degree of condensation of its inorganic network. Notably, the laser flash photolysis, fluorescence, and electron paramagnetic resonance spin-trapping (EPR ST) experiments demonstrated that TXS could react with Iod through an electron transfer reaction through its excited states, leading to the formation of radical initiating species. Interestingly, the TXS/Iod was demonstrated to be an efficient photoinitiating system for free-radical (FRP) and cationic (CP) polymerization under LEDs@385, 405, and 455 nm. In particular, whatever the epoxy monomer mixtures used, remarkable final epoxy conversions were achieved up to 100% under air. In this latter case, we demonstrated that both the photoinduced sol–gel process (hydrolysis of trimethoxysilane groups) and the cationic photopolymerization occurred simultaneously.

## 1. Introduction

Photopolymerization provides several economic advantages over the usual thermal approaches, namely rapid polymerization reactions, low energy requirements, and non-polluting and solvent-free formulations, among others [1,2,3]. Such a technique uses UV, visible, or NIR light to initiate chemical reactions generating new polymeric/composite materials. Among various factors conditioning the efficiency of the photopolymerization process in coating applications, photoinitiators have been recognized to play a crucial role, as they enable tuning of the curing speed, the tack-free index, and the mechanical properties of the resulting polymer or composite materials [2,4]. However, the use of UV-absorbing photoinitiators/photosensitizers has raised some toxicity issues and prompted researchers to synthesize new red-shifted absorbing photoinitiating systems. To date, various photoinitiators have been designed to extend their absorbance spectra in the visible or IR range [4,5,6,7,8,9,10,11,12,13,14]. This stems from the fact that visible or IR light is not only safer than UV light but also presents a higher penetration capability, despite the use of UV-absorbing monomers, substrates, and pigments. Among miscellaneous photoinitiating systems investigated in the field of photochemistry, thioxanthone (TX) and its derivatives [5,15,16,17,18] have been widely employed because of their high initiation efficiency under light irradiation and their interesting absorption characteristics in the near-UV and visible light domains [15,16,19,20,21,22,23,24]. In most cases, the initiating free radicals derived from TX derivatives are produced by the hydrogen abstraction of TX in its excited states when combined with hydrogen donors [24,25,26]. In this investigation, a multifunctional visible-light TX-based photosensitizer bearing a siloxane moiety (TXS) was designed and synthesized. Such a group would enable the incorporation of a TX photosensitizer into a siloxane network by photoinduced sol–gel chemistry. Such a multifunctional photosensitizer has not yet been described in the literature. The first part of this work is devoted to synthesizing and characterizing the multifunctional TX derivative by steady-state photolysis, fluorescence, phosphorescence, laser flash photolysis, cyclic voltammetry, and electron paramagnetic resonance spin-trapping (EPR ST) experiments. The second part of this study consists of demonstrating the photoinduced sol–gel reactions by liquid ^1^H and solid-state ^29^Si NMR experiments in which TXS is combined with a diaryliodonium salt (Iod) under LED irradiation. The capability of this new photoinitiating system, i.e., TXS/Iod, to promote free-radical and cationic photopolymerization of siloxane/epoxy-based monomer mixtures is finally demonstrated by Real-Time Fourier Transform InfraRed spectrometry (RT-FTIR) under LEDs@ 385, 405 and 455 nm, under air and in laminate conditions.

## 2. Results and Discussion

### 2.1. Synthesis of TXS

**Scheme 1:**
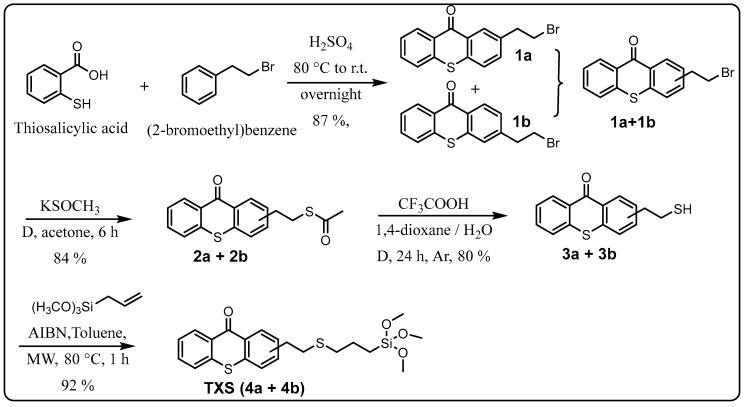
Global procedure for the synthesis of TXS.

In order to reach the first objective of incorporating the thioxanthone into a siloxane network by photoinduced sol–gel chemistry, a thioxanthone-based visible-light photosensitizer containing a trimethoxysilane moiety was designed and synthesized in four steps, as shown in Scheme 1. The first step consisted of reacting 2-bromoethylbenzene with thiosalicylic acid in the presence of sulfuric acid to yield 2-(2-bromoethyl)thioxanthone (via cyclization) as a mixture of two isomers (**1a**/**1b**) with an 87% yield. The mechanism for the formation of **1b** is described in Scheme S1 (Supplementary Materials). The formation of **1a** can also be described similarly, just by modifying the position of the electrophilic aromatic substitution (step 3): the meta position instead of the para one. The reaction would probably follow a Friedel–Crafts acylation (steps 1–5), followed by a keto–enol tautomerism (steps 6–7), and finally, oxidation driven by the aromatization (stable system) promoted by concentrated H_2_SO_4_ (steps 7–8) to yield the final product. The electrophilic aromatic substitution (step 3) could occur at three different reactive sites on (2-bromoethyl)benzene, namely the ortho, para, and meta positions, but preferentially at ortho and para due to the slightly positive inductive effect (I+) of the 2-bromoethyl substituent. However, the structural characterization of the final products showed the absence of ortho product (probably due to the steric hindrance of the 2-bromoethyl moiety) while a mixture of both para product (**1b**) and meta product (**1a**) was isolated. The mixture of two isomers, **1a** and **1b,** cannot be separated by TLC. ^1^H NMR analyses show that the isolated products (**1a** + **1b**) are quite pure: it is important to note that the small peaks around 3.5 ppm come from the meta product but not from impurities.

The introduced bromide group was converted into a thioester function with an 84% yield just by reacting with potassium thioacetate. The resulting TX thioester, **2a**/**2b,** was subjected to an acidic hydrolysis leading to a thiolated thioxanthone as a mixture of two regioisomers (**3a**/**3b**) with an 80% yield. Due to the presence of two isomers, 3a and 3b, several peaks can be found in its ^1^H-NMR. Large peaks can be attributed to protons of the major isomer, while small peaks nearby are attributed to the similar protons of the minor isomer. This tendency can also be observed in the previous mixture of isomers, i.e., (**1a** + **1b**) and (**2a** + **2b**). Finally, the generated reactive -SH function on **3a**/**3b** underwent a click thiol-ene reaction with allyltrimethoxysilane under microwave (MW) irradiation to obtain the targeted photosensitizer bearing a trimethoxysilane moiety TXS (**4a**/**4b)** in a nearly quantitative yield (92%).

**Scheme 2:**
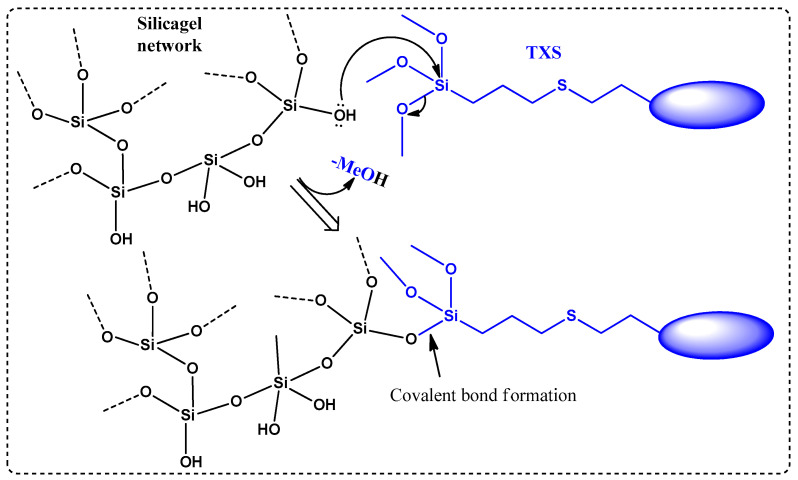
Covalent bond formation between siloxane network and TXS via nucleophilic substitution.

It is worth mentioning that microwave (MW) irradiation enabled not only the acceleration of the conversion rate (only 1 h) but also avoided the formation of side products. Indeed, less than 10% of conversion into the desired product along with many different side products were observed when an identical reaction (same temperature, same concentration of reagents) was performed under classical heating conditions (no MW irradiation) despite a much longer reaction time (16 h). Furthermore, the presence of a trimethoxysilane moiety on the final product highly complicated the final purification step: the conventional chromatography on silica gel was not suitable because the desired product could not migrate out, even through a very short silica gel column (less than 3% of TXS was isolated). This loss of product could be explained as follows: -OH groups on silica gel undergo a nucleophilic substitution with trimethoxysilane moiety presented on the structure of TXS to liberate volatile MeOH molecules, as described in Scheme 2. The resulting covalent bonds, Si-O, between the silica gel network and TXS would prevent the elution of the latter out of the column. The interaction between silanol groups in the silica gel with the trimethoxysilane is well-known in the literature [27]. After several attempts at purification by other techniques (precipitation, extraction) using different solvents, pure **4a**/**4b** was isolated with a 48% yield only by trituration in cold MeOH. This yield can be considered relatively high because of the partially soluble nature of this product, even in cold MeOH.

### 2.2. Photoreactivity of the TXS/Iod Photoinitiating System

**Figure 1:**
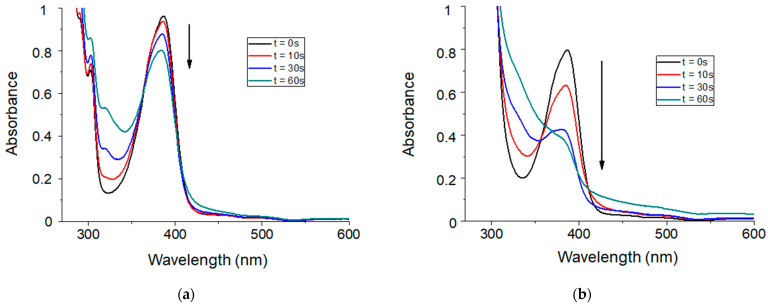
Steady-state photolysis in chloroform during 60 s of irradiation under LED@405 nm (100 mW/cm^2^) of (**a**) TXS alone and (**b**) TXS/Iod photoinitiating system. [TXS] = 4.3 × 10^−3^ mol/L, and [Iod] = 8.6 × 10^−3^ mol/L.

The reactivity of thioxanthone-based molecules as photosensitizers of iodonium salts has been previously described in the literature. Several spectroscopic studies have reported a photoredox reaction between thioxanthone and iodonium salts. However, and to the best of our knowledge, the addition of a siloxane moiety to thioxanthone has not yet been reported. Therefore, the overall photochemical reactivity of TXS was first demonstrated by steady-state photolysis in chloroform. The UV-absorption spectrum of TXS is described in Figure S1. The absorption of TXS ranges from 350 to 500 nm, with a maximum absorption at ~390 nm and ε _383nm_ equal to 7160 cm^−1^ M^−1^. Its spectrum is slightly red-shifted in comparison with the unmodified TX spectrum. The absorbance range of TXS fully merges with the emission spectra of LEDs@385 nm, @405 nm, and @455 nm, which could make TXS an interesting visible-light photosensitizer of Iod to promote cationic photopolymerization. For this purpose, the photolysis of TXS in combination with Iod was evaluated. Figure 1 describes the evolution of the UV-visible absorbance of TXS upon visible-light exposure with and without the addition of an electron acceptor molecule i.e., Iod. After 60 s of irradiation, a slight decrease in the TXS absorbance alone is observed. On the contrary, the UV-vis absorbance of the TXS strongly decreases in the same conditions when Iod is introduced. This result agrees with a strong synergic interaction between TXS and Iod, and it is expected that a TXS/Iod photoinitiating system could be highly reactive to perform cationic photopolymerization.

**Figure 2:**
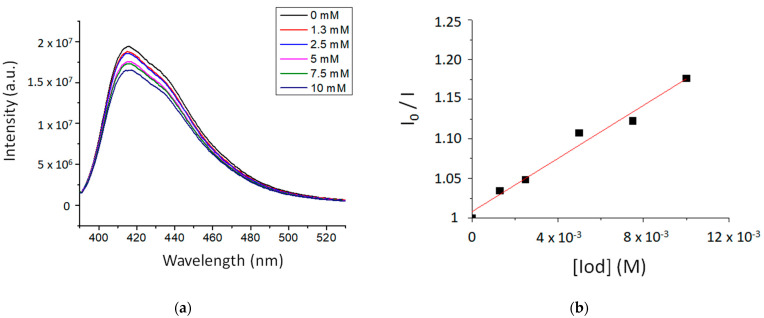
(**a**) Fluorescence emission spectra of TXS in CHCl_3_ with the gradual addition of Iod and (**b**) The Stern–Volmer plot describing the fluorescence quenching of ^1^TXS by Iod. λ_excitation_ at 385 nm.

**Figure 3:**
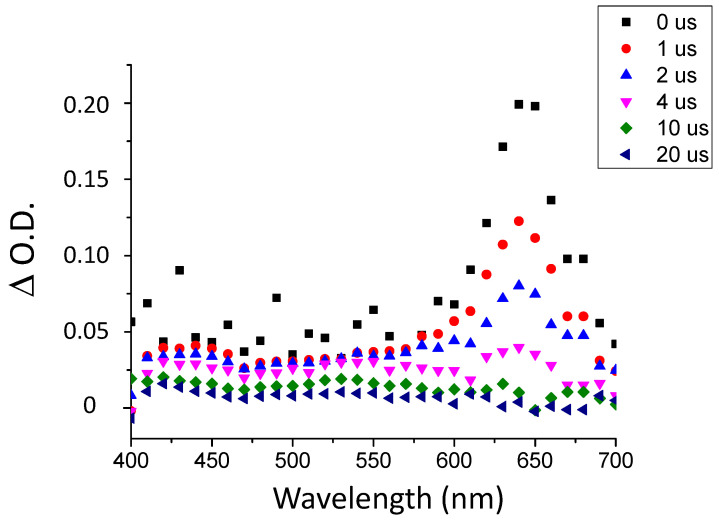
Transient absorption spectrum of TXS in chloroform under argon atmosphere after laser excitation at λ_355 nm_.

**Figure 4:**
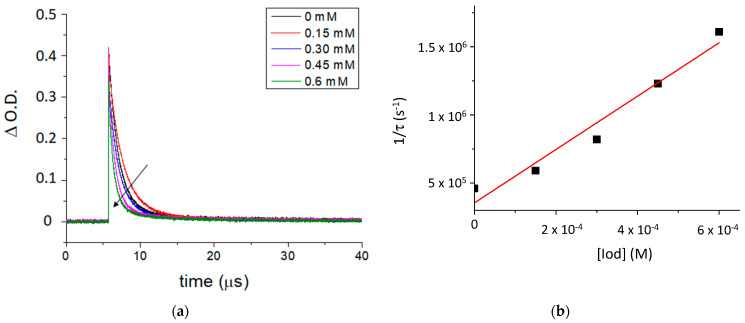
(**a**) Quenching of the transient absorption triplet state of TXS by an increasing concentration of Iod at 640 nm, and (**b**) the Stern–Volmer plot of ^3^TXS quenching by Iod. Laser excitation at λ_355 nm_.

The fluorescence properties of TXS enabled the evaluation of the possibility of interaction between the TXS singlet excited states and Iod under light activation. The fluorescence quantum yield of TXS is 0.013. The fluorescence of TXS at 415 nm is quenched when the concentration of Iod in the medium increases (Figure 2), and the fluorescence quenching constant, k_q_τ_0_, was evaluated to be equal to 16.8 M^−1^. This supports an electron transfer reaction between Iod in its ground state and ^1^TXS* (singlet excited state). The negative ΔG_S_ of ^1^TXS/Iod (−1.38 eV) was estimated by the Rehm–Weller equation [28] (Equation (S1)) using an oxidation potential of TXS (E_ox, TXS_ = 1.03 V, Figure S2), reduction potential of Iod, E_Red, Iod_ = −0.71 V vs. SCE [29,30] and the TXS excited singlet state energy (E_S, TXS_ = 3.12 eV, fluorescence experiments). The negative value of ΔG_S_ is consistent with a possible electron transfer reaction from the TXS singlet state to the Iod. The electron transfer quantum yield, φ_eT_ from ^1^TXS to Iod, and deducted from φ_eT_ = k_q_τ_0_ [Iod]/(1+ k_q_τ_0_ [Iod]) (with [Iod] = 3.7 × 10^−2^ M), is evaluated to be equal to 0.38. This latter value highlights the formation of a high quantity of radical species favoring the initiation of photopolymerization. In the same manner, the ΔG_T_ from the ^3^TXS/Iod system is also favorable and is evaluated at −0.96 eV (with E_T, TXS_ = 2.70 eV according to the phosphorescence experiment, Figure S3). The arising question now is whether ^1^TXS* undergoes an intersystem crossing. The TXS triplet quantum yield was evaluated to be equal to 0.57; this value highlights that an intersystem crossing is favorable between ^1^TXS* and ^3^TXS*. According to the transient absorption spectrum of TXS, an excited triplet state is observed at 640 nm and shows a lifetime of 2 μs (Figure 3). The increasing addition of Iod leads to a decrease in the TXS triplet lifetime, which allows the determination of the quenching rate constant, k_q_ (^3^TXS/Iod), of the triplet excited state of TXS by the Iod. Interestingly, the kq (^3^TXS/Iod) was evaluated at 2 × 10^9^ M^−1^ (Figure 4), whereas the kq (^3^TXS/O_2_) is equal to 1 × 10^9^ M^−1^ (Figure S4). Therefore, TXS/Iod can be considered an interesting photoinitiating system to promote photopolymerization under air.

### 2.3. Photoinduced Sol–Gel Reaction with TXS/Iod System

**Figure 5:**
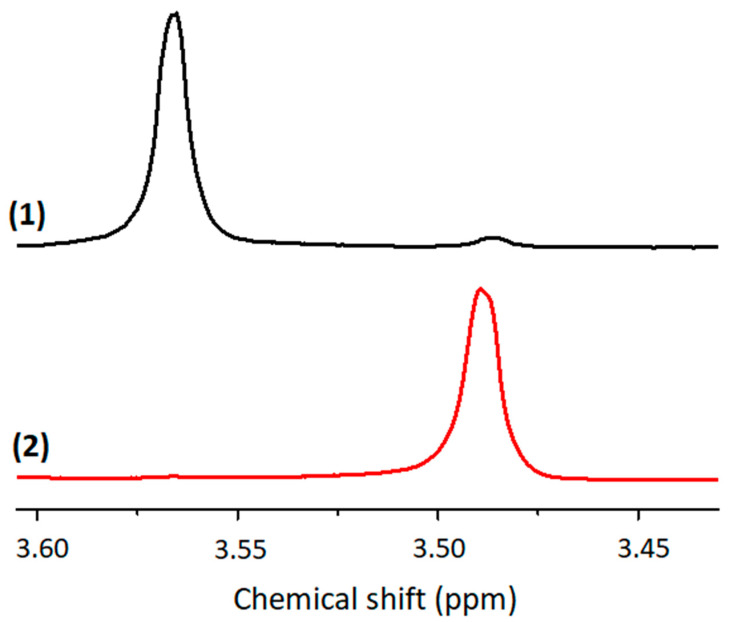
Liquid ^1^H NMR of the TXS/Iod solution in CDCl_3_ before (**1**) and after (**2**) LED@385 nm irradiation (10 min, 130 mW/cm^2^).

**Figure 6:**
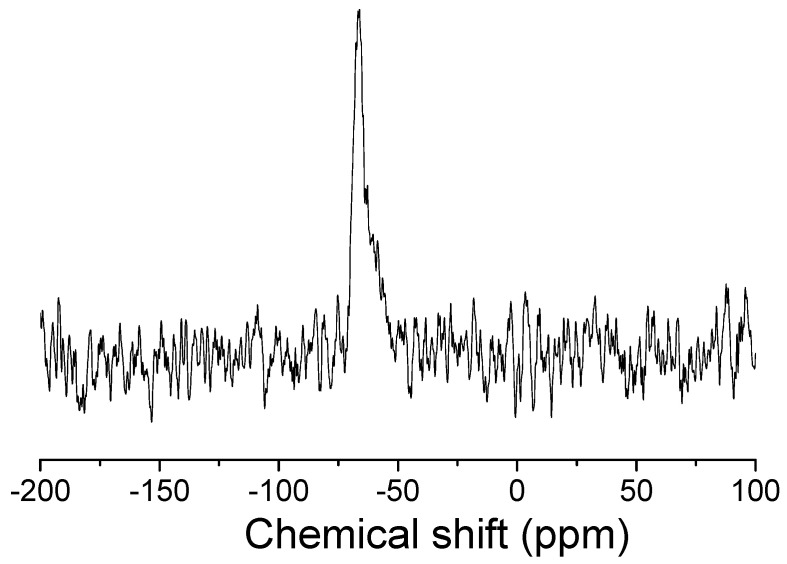
Solid ^29^Si NMR of the TXS/Iod/DPDO material after 15 min of LED@405 nm irradiation (100 mW/cm^2^).

Interestingly, the irradiation of the TXS/Iod solution leads to a complete disappearance of the ^1^H signal at 3.56 ppm, which is due to the methyl protons of the methoxysilane groups on the TXS backbone (Si-OCH_3_). Subsequently, a new ^1^H signal due to the methyl protons of methanol is observed at 3.49 ppm, thus perfectly confirming the hydrolysis/condensation of the trimethoxysilane of the TXS (Figure 5). Additional NMR experiments confirmed this observation: indeed, ^29^Si NMR experiments were performed on the solid material resulting from the irradiation of the TXS/Iod/DPDO formulation. In order to perform the solid ^29^Si NMR experiments, the photoinduced material generated from the irradiation of the TXS/Iod/monomer formulation should be a powder. Therefore, the use of a difunctional monomer, i.e., DPDO, is perfectively adapted to obtain this kind of solid material, as the epoxy conversion reaches 80% under LED@405 nm under air. The use of a monofunctional monomer does not allow for obtaining a solid material under LED@405 nm. It should be noticed that TXS is the only molecule possessing trimethoxysilane groups that could be involved in a photoinduced sol–gel reaction. After the LED@405 nm exposure, only one ^29^Si signal is observed at −66 ppm and is attributed to the T3 backbone (Figure 6). Both liquid and solid ^29^Si NMR results undeniably confirm the formation of photoacids (resulting from the photolysis of TXS/Iod), which are responsible for the photoinduced sol–gel hydrolysis and condensation of the trimethoxysilane groups of TXS. In light of the EPR, fluorescence, and NMR results, TXS can therefore be used as a photosensitizer of Iod for cationic photopolymerization (CP) and free-radical photopolymerization (FRP) and could reasonably be integrated into a hybrid sol–gel matrix without being released.

### 2.4. FRP of MAPTMS with the (TXS/Iod) Photoinitiating System

**Figure 7:**
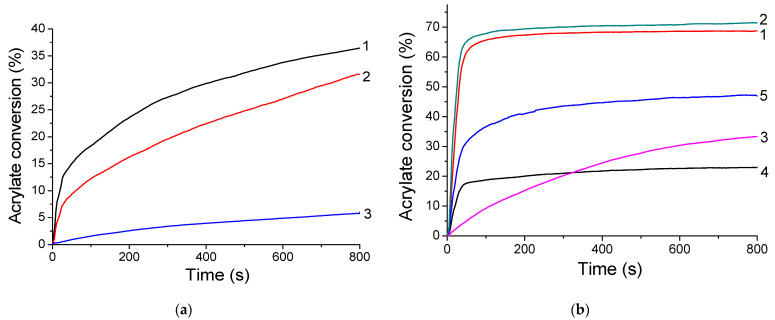
Kinetic profiles of the FRP of (**a**) MAPTMS and (**b**) MAPTMS 25 wt%/SOA 75 wt% blend mixtures using TXS/Iod (0.5%/2% *w*/*w*) as the photo-initiating system in laminate and under LED@385 nm (1), LED@405 nm (2), and LED@455 nm (3) after 800 s of irradiation. Photopolymerization under air: curve 4 = irradiation under LED@385 nm and curve 5 = irradiation under LED@405 nm.

The TXS/Iod photoinitiating system is first used for the FRP of 3-(Trimethoxysilyl)propyl methacrylate (MAPTMS) under visible-light irradiation. The kinetic profiles of MAPTMS are described in Figure 7. The final acrylate conversions reach around 30% when the formulation is irradiated under LEDs@385 and 405 nm; however, it drastically decreases to 6% under LED@455 nm irradiation under laminate. This could be explained by the low absorbance of TXS at 455 nm in comparison with that at 385 and 405 nm. The FRP of MAPTMS is observed as efficient initiating free radicals, i.e., (4-methyl)phenyl radicals, are produced under light irradiation of TXS/Iod photoinitiating system. Indeed, the generation of (4-methyl)phenyl radicals in TXS/Iod/benzene/argon solutions was also demonstrated by using 2,2-Dimethyl-4-phenyl-2H-imidazole 1-oxide (DMPIO) or *N*-*tert*-Butyl-α-phenylnitrone (PBN) spin-trapping molecules, suitable for the observation of carbon-centered radicals (Figure S5). The total 100% of the EPR signals correspond to the DMPIO (Figure S5a) or PBN (Figure S5b)-adduct with (4-methyl)phenyl radical. The (4-methyl)phenyl radical added to the DMPIO molecule has the following spin-Hamiltonian parameters, a_N_ = 1.412 mT, a_H_ = 1.888 mT, and g = 2.0059; and the PBN-adduct with (4-methyl)phenyl radical is observed with the hyperfine coupling constants, a_N_ = 1.451 mT, a_H_ = 0.227 mT, and g = 2.0061 [31]. It is also interesting to note that sulfur-centered radicals are also generated. DMPO is a suitable spin-trapping agent to trap carbon- or sulfur-centered radicals [31]. Indeed, the irradiation of benzene solutions containing TXS/Iod/DMPO/benzene under inert atmospheres (Figure S6) resulted in the immediate generation of characteristic six-line EPR signal of ^∙^DMPO-(4-methyl)phenyl adduct (55%) with the hyperfine coupling constants, *a*_N_ = 1.401 mT, *a*_H_^β^ = 1.967 mT; and *g* = 2.0060, along with the superimposed signal assigned to the DMPO-adduct with a sulfur-centered radical [32] (^∙^DMPO-SR, 45%) with the hyperfine coupling constants, *a*_N_ = 1.359 mT, *a*_H_^β^ = 1.149 mT, *a*_H_^γ^ = 0.084 mT, and *a*_H_^γ^ = 0.025 mT. Unfortunately, the FRP of MAPTMS does not work under air, as the triplet state of TXS is quenched by O_2,_ and (4-methyl)phenyl radicals can react with O_2_ to produce peroxyl radicals, which are inert toward FRP [33]. Interestingly, the addition of SOA, a multiple functional acrylate monomer, to the MAPTMS formulation leads to an increase in the final acrylate conversions up to 70% under LEDs@385 and 405 nm, and to 35% under LED@455 nm in laminate (Figure 7b). The addition of SOA not only increases the final conversion in laminate experiments but also allows FRP under air. The high viscosity of SOA limits the oxygen diffusion into the acrylate blend mixture, thus avoiding the formation of peroxyl radicals.

### 2.5. Cationic Photopolymerization and Hydrolysis/Condensation of GPTMS/Epoxy Monomers with the (TXS/Iod) Photoinitiating System

**Figure 8:**
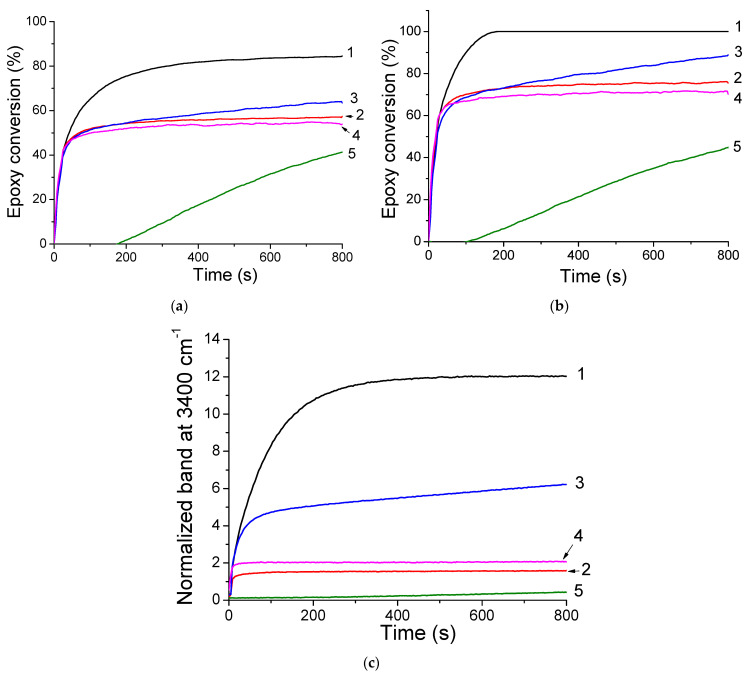
Evolution of the epoxy conversions of (**a**) DPDO and (**b**) GPTMS in the DPDO/GPTMS mixture, and (**c**) hydrolysis extent vs. irradiation time of GPTMS 25 wt%/DPDO 75 wt% formulation with TXS/Iod (1%/2% *w*/*w*) at LED@385 nm (1) under air, (2) in laminate at LED@405 nm, and (3) under air, (4) in laminate, and (5) at LED@455 nm under air.

**Figure 9:**
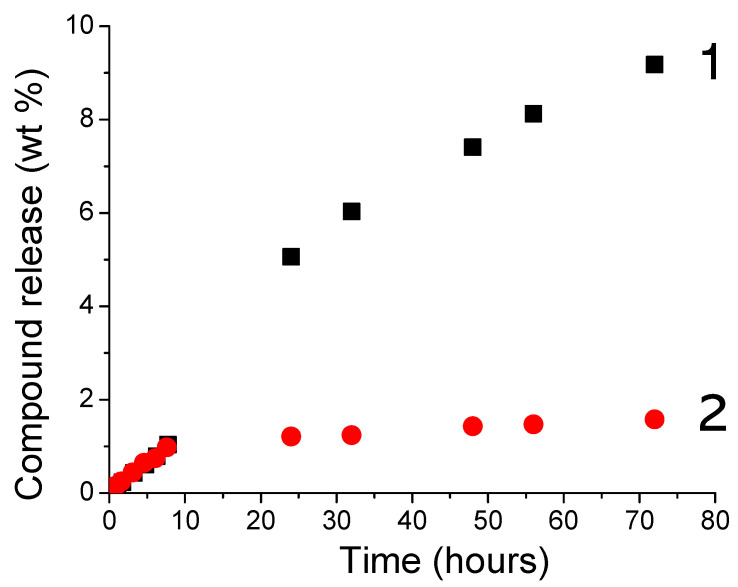
Release of TX (**1**) and TXS (**2**) from the photoinduced-GPTMS 25wt%/EPOX 75 wt% materials after 15 min under LED@405 nm irradiation (100 mW/cm^2^).

In a second experiment, with the idea to develop new visible-cured organic-inorganic hybrid materials, TXS/Iod was used as a photoinitiating system to promote the concomitant polymerization of the organic and inorganic phases by the photogenerated Brönsted acids. This reaction was first observed by Crivello et al. [34] in 1995, who described the rapid UV polymerization of the epoxy groups followed by a slow consumption of the trialkoxysilyl groups by hydrolysis/condensation of a monomer bearing both the epoxycyclohexyl and trimethoxysilyl reactive functions with a well-known cationic triarylsulfonium catalyst. Subsequently to this investigation, some other studies carried out by Crivello [35,36], Soucek [37,38,39,40], Sangermano [41,42,43,44,45], and Chemtob [46,47,48] have described such a phenomenon with the use of aryliodonium or arylsulfonium salts associated with several organic-inorganic hybrid monomers comprising an organic resin and various epoxy or vinylether alkoxysilane. Figure 8 shows the RT-FTIR kinetic profiles of dipentene dioxide (DPDO) and (3-Glycidyloxypropyl)trimethoxysilane (GPTMS) in the DPDO/GPTMS mixture and the hydrolysis extent vs. irradiation time of the same formulation in the presence of TXS/Iod (1%/2% *w*/*w*) upon different LEDs irradiation. The other kinetics profiles are displayed in Supporting Information (Figures S7–S11). Table 1 summarizes the final epoxy conversions of DPDO, GPTMS, and 3,4-Epoxycyclohexylmethyl 3,4-epoxycyclohexanecarboxylate (EPOX) after 800 s of irradiation under LEDs@385, 405, and 455 nm. Interestingly, higher epoxy conversions are observed in air compared with laminate conditions. These results can be explained by a synergetic effect between the polymerization of both the organic and inorganic parts. While epoxy groups disappear upon LED irradiation, a new band at 3400 cm^−1^ attributed to the photoacid-catalyzed hydrolysis of the SiOMe groups arises and leads to the formation of silanols. This phenomenon is more significant when cationic photopolymerization occurs under air. Indeed, in non-laminated samples, air moisture may diffuse throughout the formulation, favoring, thus, the photoacid-catalyzed hydrolysis of the SiOMe groups. As demonstrated by Penczek and Kubisa et al. [49,50], hydroxyl groups can be involved in the cationic photopolymerization process. Indeed, during the cationic propagation step, a nucleophilic attack from hydroxyl groups to the tertiary oxonium ion end of the polyether chains occurs, yielding a protonated ether. The new propagating oxonium species obtained through proton transfer reactions can thus initiate new polymer chain growth. The low final epoxy conversions at 455 nm are associated with the low absorbance of TXS and TX at this wavelength. Interestingly, we also demonstrate that the addition of the trimethoxysilane group on the TX structure limits its diffusion throughout the photoinduced materials (less than 2%), whereas the TX is released five times more in the same conditions (Figure 9). Indeed, as demonstrated by solid-state ^29^Si NMR spectroscopy, the irradiation of TXS/Iod leads to the sol–gel hydrolysis and condensation of the trimethoxysilane groups of the TXS. During the cationic photopolymerization of EPOX/GPTMS mixture, the siloxane moiety of TXS subsequently anchors to the siloxane network of GPTMS, thus avoiding its release.

## 3. Materials and Methods

### 3.1. Materials

The (3-Glycidyloxypropyl)trimethoxysilane (GPTMS), 3-(Trimethoxysilyl)propyl methacrylate (MAPTMS), dipentene dioxide (DPDO), (3,4-epoxycyclohexane)methyl 3,4-epoxycyclohexylcarboxylate (EPOX), thioxanthone (TX), soybean oil acrylate (SOA), and *bis*(4-methylphenyl)iodonium hexafluorophosphate (98%) were purchased from Sigma-Aldrich. The 5,5-dimethyl-1-pyrroline *N*-oxide (DMPO, distilled prior to the application), *N*-tert-butyl-α-phenylnitrone (PBN), and nitrosodurene (ND) were provided by Sigma-Aldrich, and the 2,2-dimethyl-4-phenyl-2H-imidazole 1-oxide (DMPIO) was purchased from Enzo Life Sciences. All the solvents were purchased from Sigma Aldrich (Saint-Quentin-Fallavier, France) and used without any further purification. Table 2 summarizes the structures of the compounds used in the investigation.

### 3.2. Light Sources

The light-emitting diodes (LEDs) at 385 (60 mW/cm^2^), 405 (100 mW/cm^2^), and 455 nm (38 mW/cm^2^) were used to initiate the CP and FRP of the epoxy and acrylate monomers, respectively. The LEDs were provided by THORLABS.

### 3.3. Steady-State Photolysis

The photolysis of the TXS-based systems was performed in chloroform at LED@405 nm exposure in an air atmosphere.

### 3.4. UV-Visible Absorption

The UV-Vis spectra were collected on a Perkin Elmer Lambda 2 UV–vis spectrophotometer between 200 and 800 nm.

### 3.5. Fluorescence Studies

Steady-state fluorescence spectra were obtained on a FluoroMax-4 spectrofluorometer and a Perkin-Elmer Lambda 2 spectrometer. The interaction rate constants, K_q_, between TXS and Iod were calculated using the Stern–Volmer equation: (I_0/_I) = 1 + K_q_[Iod], where I_0_ and I represent the fluorescence intensity of TXS without and with the addition of Iod.

### 3.6. Phosphorescence Studies

The phosphorescence spectra were recorded using a FluoroMax-4 spectrofluorometer equipped with an Xe-pulsed lamp. Luminescence measurements were performed in a glassy matrix of ethanol at 77 K. The sample was placed in a 5 mm diameter quartz tube inside a Dewar filled with liquid nitrogen.

### 3.7. Laser Flash Photolysis Experiments

Laser flash photolysis experiments were performed in an argon atmosphere at room temperature according to a previously described procedure [51]. Analyses were carried out on a 7 × 10^−5^ M solution of TXS in chloroform.

### 3.8. Triplet Quantum Yield (Φ_T_)

The triplet state quantum yield, Φ_T_, of TXS was determined according to a previously described method [51] in chloroform, using a comparative method with TX with the following equation:

$$\Phi_{T}=\Phi_{T}^{Std}\frac{∆A_{T}\varepsilon_{T}^{Std}}{{∆A}_{T}^{Std}\varepsilon_{T}}$$

where ΔAT and ${∆A}_{T}^{Std}$ are the changes in the triplet state absorbance of TXS and TX, respectively; ԑ_T_ and $\varepsilon_{T}^{Std}$ are the molar extinction coefficients for the triplet states of TXS and TX, respectively; and $\Phi_{T}^{Std}$ is the triplet quantum yield of the reference TX ($\Phi_{T}^{Std}$ = 0.56).

### 3.9. Electron Paramagnetic Resonance (EPR)

The solutions were prepared in benzene under an argon atmosphere and irradiated at RT directly in the EPR resonator using an LED@385 nm according to a previously described procedure [52]. When the presence of oxygen was required in the experimental systems, the spin-trapping experiments were performed in solutions partially saturated by argon in order to eliminate a significant EPR line broadening caused by the high solubility of O_2_ in benzene [53]. Simulations of the experimental EPR spectra were performed by Bruker software, WinEPR (Billerica, MA, USA), and the EasySpin toolbox [54].

### 3.10. Cyclic Voltammetry

The oxidation potential of TX and TXS was determined in ACN according to a previously described method [6]. Briefly, a standard three-electrode cell configuration (vitreous working carbon electrode, a saturated calomel electrode as a reference, and a gold wire as counter electrode) using an AUTOLAB potentiometer/galvanometer employing GPES electrochemical software version 4.9 (Utrecht, The Netherlands) was employed to evaluate the oxidation potential (E_ox_) of thioxanthone derivative. The measurement was performed in acetonitrile with tetrabutylammonium hexafluorophosphate ([NEt_4_PF_6_] = 0.1 M in ACN) as the supporting electrolyte. The thioxanthone derivative solution was prepared at 10^−3^ M in this supporting electrolyte. The oxidation potential was measured between 0 and 2 V with a scan rate of 0.05 V.s^−1^.

### 3.11. Photopolymerization Investigations

The reactivity of the acrylate and epoxy formulations was followed by Real-Time Fourier Transform InfraRed Spectroscopy (RT-FTIR, JASCO FTIR 4700) at 3050 cm^−1^ for GPTMS, 850 cm^−1^ for DPDO, at 790 cm^−1^ for EPOX, and at 1637 cm^−1^ for MAPTMS and SOA, according to previous experiments [6].

### 3.12. Extraction Study

Pellets of 50 mg were prepared from formulations of GPTMS/EPOX (25%/75% *w*/*w*) in the presence of TX/Iod (1%/2% *w*/*w*) or TXS/Iod (1%/2% *w*/*w*). Each pellet was cured under LED@405 nm (100 mW·cm^−2^) for 15 min in a 1.1 cm × 1.1 cm round silicon mold to obtain tack-free samples. Each pellet was then immersed in 10 mL of ethanol. Then, 0.5 mL was collected and diluted four times to collect the UV-visible spectra at regular intervals. The experiments were repeated in triplicate for each system.

### 3.13. ^29^Si Solid-State NMR

A TXS/Iod/DPDO (33%/17%/50% *w*/*w*) sample was photopolymerized under LED@405 nm (100 mW/cm^2^) for 15 min to obtain a solid sample. Its ^29^Si NMR spectrum was obtained using a Bruker Advance III NMR spectrometer, with a ^29^Si Larmor frequency of 79.5 MHz. A 4 mm double-resonance MAS probe was used, with a MAS frequency of 10 kHz. ^1^H/^29^Si cross-polarization (CP) at 90° and a pulse length of 3.25 ms, a ^1^H spin-lock field of 70 kHz (2.9 dB), and a contact time of 6 ms permitted to obtain the ^29^Si spectrum. The ^1^H dipolar decoupling field intensity during acquisition was set to 8.5 dB, and the recycle delay to 6 s. The spectra were referenced with the ^29^Si NMR peak of hectorite (96.0 ppm with respect to TMS). A total of 85,000 scans were carried out, and spectra were recorded at 23 °C.

### 3.14. Synthesis of (2-Bromoethyl)thioxanthone (***1a*** + ***1b***)

**Scheme 3:**
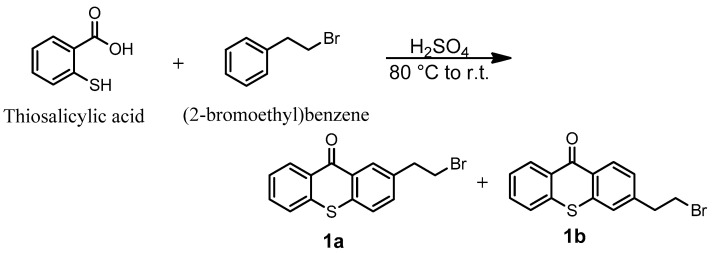
Synthesis of (2-Bromoethyl)thioxanthone (**1a** + **1b**).

The synthesis of (2-Bromoethyl)thioxanthone is described in Scheme 3. The synthesis procedure is as follows: 15 mL of concentrated sulfuric acid was slowly added to thiosalicylic acid (1.6 g, 10.3 mmol). The solution was stirred at RT until a transparent solution was obtained (about 15 min), and then (2-bromoethyl)benzene (6 mL, 44.4 mmol) was added dropwise over 30 min. After 1 h, the solution was heated to 80 °C for 2 h (observation: after only 5 min heating, the solution became red) and then stirred for a further 15 h at RT before being slowly poured into water (150 mL). The mixture was filtered, and the obtained yellow solid was washed with water (200 mL), then dissolved in dichloromethane (100 mL), dried with MgSO_4_, and filtered off. The filtrate was evaporated under reduced pressure, yielding 2.86 g of thioxanthones (**1a** + **1b**) as a yellow solid (87% yield).

^1^H NMR (400 MHz, CDCl_3_, Figure S12) δ (ppm): 8.69 (d, *J* = 8.0 Hz, 1H, H_1_), 8.47 (s, 1H, H_7_), 7.60–7.46 (m, 5H, H_2_ + H_3_ + H_4_ + H_5_ + H_6_), 3.65 (t, *J* = 6.0 Hz, 2H, H_9_), 3.30 (t, *J* = 6.0 Hz, 2H, H_8_). ^13^C-NMR (400 MHz, CDCl_3_, Figure S13) δ (ppm): 180.21, 137.42, 135.97, 133.91, 132.46, 130.07, 129.71, 126.45, 126.20, 38.93, 32.85. FTIR (cm^−1^, Figure S14): 3054 (ν_C-H, aromatic_), 2959 (ν_asym_ CH_2_), 1632 (strong, ν_C=O_), 1588 (strong, ν_C=C, aromatic_), 736 (strong, ν_C-S_), 629 (strong, ν_C-Br_).

### 3.15. Synthesis of (2-Ethylthioacetate)thioxanthone (***2a*** + ***2b***)

**Scheme 4:**
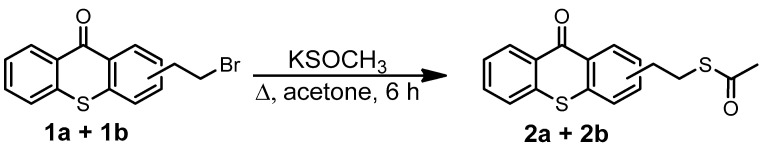
Synthesis of (2-Ethylthioacetate)thioxanthone (**2a** + **2b**).

The synthesis of (2-Ethylthioacetate)thioxanthone is described in Scheme 4. The synthesis procedure is as follows: potassium thioacetate (1.07 g, 9.40 mmol) at RT was added to a solution of (2-Bromoethyl)thioxanthone (1.57 g, 4.92 mmol, **1a** + **1b**) in 15 mL of anhydrous acetone. The mixture was refluxed in the dark for 6 h under argon. After cooling to RT, the acetone was evaporated. Water (25 mL) was added to the crude material, and the mixture was extracted with CH_2_Cl_2_ (2 × 50 mL). The organic phases were combined and dried with Na_2_SO_4_. The solution was evaporated and dried under vacuum at RT, resulting in 1,3 g thioxanthone (**2a** + **2b**) as a brown solid (84% yield).

^1^H NMR (400 MHz, CDCl_3_, Figure S15) δ (ppm): 8.56 (d, *J* = 8.0 Hz, 1H, H_1_), 8.40 (s, 1H, H_7_), 7.55–7.42 (m, 5H, H_2_ + H_3_ + H_4_ + H_5_ + H_6_), 3.15 (t, *J* = 6.0 Hz, 2H, H_8_), 2.96 (t, *J* = 8.0 Hz, 2H, H_9_), 2.31 (s, 3H, H_10_). ^13^C-NMR (400 MHz, CDCl_3_, Figure S16) δ (ppm): 195.47 (C_16_), 179.88 (C_7_), 138.35 (C_12_), 137.26 (C_6_), 135.39 (C_9_), 133.01 (C_4_), 132.21 (C_11_), 129.87 (C_10_), 129.41(C_13_), 129.18 (C_2_), 129.14 (C_1_), 126.23 (C_3_ + C_8_), 125.99 (C_5_), 35.39 (C_14_), 30.67 (C_15_), 30.18 (C_17_). FTIR (cm^−1^, Figure S17): 3055 (ν_C-H, aromatic_), 2930 (ν_asym_ CH_2_), 1686 (strong, ν_C=O_, _thioester_), 1632 (strong, ν_C=O_), 1588 (strong, ν_C=C, aromatic_), 735 (strong, ν_C-S_).

### 3.16. Synthesis of (2-Mercaptoethyl)thioxanthone (***3a*** + ***3b***)

**Scheme 5:**
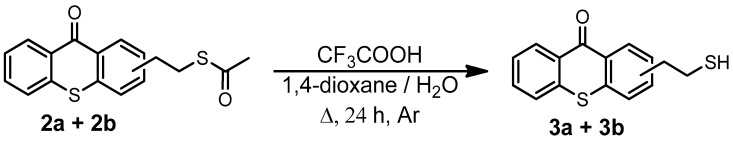
Synthesis of (2-Mercaptoethyl)thioxanthone (**3a** + **3b**).

The synthesis of (2-Mercaptoethyl)thioxanthone is described in Scheme 5, and the synthesis procedure is described in ref. [55]. The yield of the thioxanthone derivative reached 80%. ^1^H NMR (400 MHz, CDCl_3_, Figure S18) δ (ppm): 8.61 (d, *J* = 4.0 Hz, 1H, H_1_), 8.45 (s, 1H, H_7_), 7.61–7.47 (m, 5H, H_2_ + H_3_ + H_4_ + H_5_ + H_6_), 3.05 (t, *J* = 8.0 Hz, 2H, H_8_), 2.85 (q, *J* = 8.0 Hz, 2H, H_9_), 1.39 (t, *J* = 8.0, 3H, H_10_). ^13^C-NMR (400 MHz, CDCl_3_, Figure S19) δ (ppm): 179.93 (C_7_), 138.28 (C_12_), 137.30 (C_6_), 135.42 (C_9_), 133.17 (C_4_), 132.28 (C_11_), 129.91 (C_10_), 129.50 (C_2_), 129.21 (C_13_), 129.18 (C_1_), 126.29 (C_8_), 126.21 (C_3_), 126.04 (C_5_), 39.75 (C_14_), 25.85 (C_15_). FTIR (cm^−1^, Figure S20): 3051 (ν_C-H, aromatic_), 2933 (ν_asym_ CH_2_), 2551 (ν_SH_), 1626 (ν_C=O_), 1586 (ν_C=C, aromatic_), 739 (strong, ν_C-S_).

### 3.17. Synthesis of 2-(2-{[3-(Trimethoxysilyl)propyl]sulfunyl}ethyl-9H-thioxanthen-9-one (TXS)

**Scheme 6:**
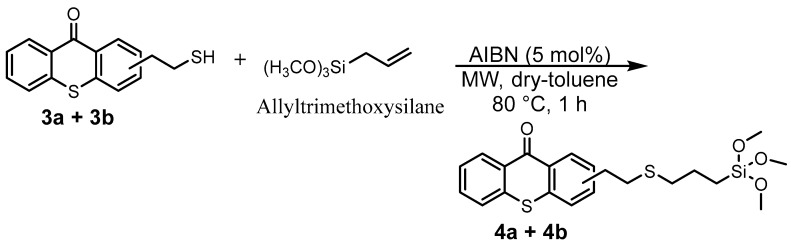
Synthesis of 2-(2-{[3-(trimethoxysilyl)propyl]sulfunyl}ethyl-9H-thioxanthen-9-one (**4a** + **4b**).

The synthesis of 2-(2-{[3-(trimethoxysilyl)propyl]sulfunyl}ethyl-9H-thioxanthen-9-one is described in Scheme 6. The synthesis procedure is as follows: A mixture of thioxanthones **3a** and **3b** (1 g, 3.67 mmol), allyltrimethoxysilane (2.92 mL, 18.3 mmol), and 2 mL of dry toluene was suspended in a microwave (MW) tube provided with a cap. Afterward, the reaction mixture was bubbled with argon for 1 h before adding AIBN (5 mol %), The reaction mixture was bubbled again with argon for 15 min before being placed into an MW reactor to perform the reaction with the following conditions: 80 °C, power of 1 W, and duration of 1 h under constant stirring. The mixture was evaporated under reduced pressure on a rotary evaporator to remove the solvent, followed by a vacuum at room temperature overnight to remove the excess allyltrimethoxysilane. According to the ^1^H NMR, the crude product was almost pure, with a quantitative yield of 92%. For performing different physicochemical characterizations, removing the traces of side products was necessary and successfully conducted by trituration in cold MeOH. Indeed, purification by column chromatography on silica gel was not suitable because the resulting product reacted with the Si gel. Remark: The duration for a complete reaction depends on the size of the MW tube, i.e., a small MW tube size (10 mL) requires 1 h reaction, while a large MW tube size (30 mL) requires 3 h.

^1^H NMR (400 MHz, CDCl_3_, Figure S21) δ (ppm): 8.58 (d, *J* = 4.0 Hz, 1H, H_1_), 8.43 (s, 1H, H_7_), 7.60–7.45 (m, 5H, H_2_ + H_3_ + H_4_ + H_5_ + H_6_), 3.55 (s, 9H, H_13_), 2.99 (t, *J* = 8.0 Hz, 2H, H_8_), 2.81 (t, *J* = 8.0 Hz, 2H, H_9_), 2.56 (t, *J* = 8.0 Hz, 2H, H_10_), 1.70 (quin, *J* = 8.0 Hz, 2H, H_11_), 0.74 (t, *J* = 8.0 Hz, 2H, H_12_). ^13^C-NMR (400 MHz, CDCl_3_, Figure S22) δ (ppm): 179.84 (C_7_), 139.00 (C_12_), 137.23 (C_6_), 135.08 (C_9_), 132.97 (C_4_), 132.12 (C_11_), 129.78 (C_10_), 129.15 (C_2_), 129.08 (C_13_), 129.05 (C_1_), 126.13 (C_8_), 126.03 (C_3_), 125.92 (C_5_), 50.49 (C_19_), 35.76 (C_16_), 35.12 (C_15_), 33.15 (C_14_), 22.91 (C_17_), 8.49 (C_18_). FTIR (cm^−1^, Figure S23): 3084 (ν_C-H, aromatic_), 2939 (ν_asym_ CH_2_), 2838 (ν_Si-OCH3_), 1635 (ν_C=O_), 1586 (ν_C=C, aromatic_), 1188 (ν_Si-C_), 1074 (strong, ν_Si-OCH3_), 740 (strong, ν_C-S_).

## 4. Conclusions

In this thorough investigation, the synthesis of TXS, a new multifunctional and visible-light-activated thioxanthone-based photosensitizer was successfully synthesized, with a good overall yield of 54%. Laser flash photolysis and fluorescence experiments demonstrated that TXS may react with an electron acceptor molecule, iodonium salt (Iod), according to an electron transfer reaction through its singlet and triplet excited states, leading to the formation of radical initiating species. Kinetic studies highlighted that this new photoinitiating system, TXS/Iod, allowed for the initiation of free-radical and cationic photopolymerization in laminate and under air after LEDs@385, 405, and 455 nm activation. In this case, remarkable final epoxy conversions were achieved (up to 100%), regardless of the epoxy monomer mixtures. By considering the cationic photopolymerization experiments in laminate and under air, we evidenced that atmospheric water plays a major role in the activation of epoxy and trimethoxysilane polymerizations. Furthermore, we demonstrated that both the photoinduced sol–gel process and cationic photopolymerization occurred simultaneously. Both the liquid ^1^H and solid ^29^Si NMR measurements undeniably confirmed the formation of photoacids (resulting from the photolysis of TXS/Iod), which promoted the photoinduced sol–gel hydrolysis/condensation of the trimethoxysilane groups of TXS, with a higher degree of condensation of its inorganic network. Interestingly, for the first time, it was demonstrated that the addition of the trimethoxysilane group to the TX structure limited its diffusion throughout the photoinduced materials (less than 2% wt), whereas TX was released five times more in the same conditions. This new type of photosensitizer could be used for the synthesis of composites in dental applications.

## Figures and Tables

**Table 1:** Final epoxy conversion of GPTMS (3050 cm^−1^), DPDO (850 cm^−1^), and EPOX (790 cm^−1^) with TXS/Iod photoinitiating system under air and in laminate at different LED irradiations ([TX] = [TXS] = 1 wt% and [Iod] = 2 wt%).

	Functions	LED@385 nm		LED@405 nm		LED@455 nm	
		Air	Laminate	Air	Laminate	Air	Laminate
(TXS/Iod)/ GPTMS 100%	Epoxy GPTMS	91%	100%	98%	100%	80%	95%
(TX/Iod)/ GPTMS 100%	Epoxy GPTMS	100%	99%	100%	82%	70%	30%
(TXS/Iod)/ GPTMS 25%/ DPDO 75%	Epoxy GPTMS Epoxy DPDO	100% 84%	75% 57%	90% 65%	70% 55%	45% 41%	np np
(TX/Iod)/ GPTMS 25%/ DPDO 75%	Epoxy GPTMS Epoxy DPDO	100% 86%	95% 65%	100% 92%	100% 59%	np np	np np
(TXS/Iod)/ GPTMS 25%/ EPOX 75%	Epoxy GPTMS Epoxy EPOX	- 91%	- 74%	- 88%	- 60%	np np	np np
(TX/Iod)/ GPTMS 25%/ EPOX 75%	Epoxy GPTMS Epoxy EPOX	- 97%	- 65%	- 93%	- 67%	np np	np np

**Table 2:** Structure of the epoxy and (meth)acrylate monomers, photosensitizer, cationic photoinitiator, and spin-trapping agents used in this investigation.

Compound	Structure
Dipentene diepoxide (DPDO)	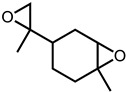
3,4-Epoxycyclohexylmethyl 3,4-epoxycyclohexanecarboxylate (EPOX)	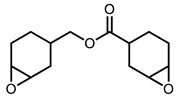
3-(Trimethoxysilyl)propyl methacrylate (MAPTMS)	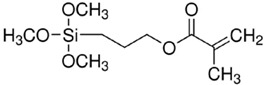
(3-Glycidyloxypropyl)trimethoxysilane (GPTMS)	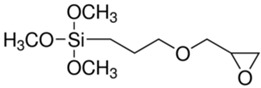
Soybean oil acrylate (SOA)	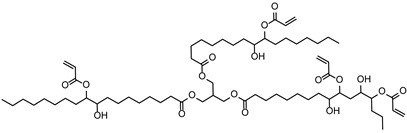
Bis(4-methylphenyl)iodonium hexafluorophosphate (Iod)	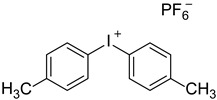
Thioxanthone (TX)	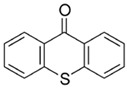
2,2-Dimethyl-4-phenyl-2H-imidazole 1-oxide (DMPIO)	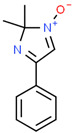
*N*-*tert*-Butyl-α-phenylnitrone (PBN)	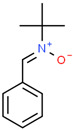

## Data Availability

The data presented in this study are available on request from the corresponding author.

## Supplementary Materials

The following supporting information can be downloaded at: https://www.mdpi.com/article/10.3390/molecules29010255/s1, Figure S1: UV-visible absorption spectra of TX and TXS in CHCl_3_; Figure S2: Cyclic voltammetry of TXS in ACN with tetrabutylammonium hexafluorophosphate ([NEt4PF6] = 0.1 M in ACN) as supporting electrolyte. The TXS solution was prepared at 10^−3^ M in this supporting electrolyte. Oxidation potential (E_Ox_) was measured between 0 and 2 V with a scan rate of 0.05 V.s^−1^; Figure S3: Normalized phosphorescence spectrum of TXS recorded in a glassy matrix of EtOH (λ_ex_. = 360 nm, delay = 2 ms, and time gate = 25 ms); Figure S4: Decay of transient absorption signal at 640 nm after the light irradiation at 355 nm under argon atmosphere (black, curve 1) and under air (red, curve 2); Figure S5: Experimental (1) and simulated (2) EPR spectra obtained during 450 s in situ exposure (LED@385 nm) of TXS/Iod in benzene solutions under argon in the presence of (a) 2,2-Dimethyl-4-phenyl-2H-imidazole 1-oxide (DMPIO) and (b) N-tert-Butyl-α-phenylnitrone (PBN) spin trapping agents. The (4-methyl)phenyl radical added to the DMPIO molecule has the following spin-Hamiltonian parameters: a_N_ = 1.412 mT, a_H_ = 1.888 mT, and g = 2.0059, and the PBN-adduct with (4-methyl)phenyl radical is observed with the hyperfine coupling constants, a_N_ = 1.451 mT, a_H_ = 0.227 mT, and g = 2.0061. In all, 100% of the signal corresponds to the DMPIO or PBN-adduct with (4-methyl)phenyl radical; Figure S6: Experimental (1) and simulated (2) EPR spectra obtained during 450 s in situ exposure (LED@385 nm) of TXS/Iod in benzene solutions under argon in the presence of DMPO spin-trapping agent. The irradiation of benzene solutions containing TXS/Iod/DMPO/benzene under an inert atmosphere resulted in the immediate generation of a characteristic six-line EPR signal of ∙DMPO-(4-methyl)phenyl adduct (55%) with the hyperfine coupling constants, a_N_ = 1.401 mT, a_Hβ_ = 1.967 mT; and g = 2.0060, along with the superimposed signal assigned to the DMPO-adduct with the sulfur-centered radical (∙DMPO-SR, 45%) with the hyperfine coupling constants, a_N_ = 1.359 mT, a_Hβ_ = 1.149 mT, a_Hγ_ = 0.084 mT, and a_Hγ_ = 0.025 mT; Figure S7: Evolution of the epoxy conversion of (1) GPTMS and (2) hydrolysis extent versus irradiation time in the presence of TX/Iod (1/2% *w*/*w*) at LED@385nm (a) under air and (b) in laminate, at LED@405nm (c) under air and (d) in laminate, and at LED@455nm (e) under air and (f) in laminate; Figure S8: Evolution of the epoxy conversion of (1) GPTMS and (2) hydrolysis extent versus irradiation time in the presence of TXS/Iod (1/2% *w*/*w*) at LED@385nm (a) under air and (b) in laminate, at LED@405nm (c) under air and (d) in laminate, and at LED@455nm (e) under air and (f) in laminate; Figure S9: Evolution of the epoxy conversion of (1) EPOX and (2) hydrolysis extent versus irradiation time in the EPOX 75%/GPTMS 25% formulation in the presence of TXS/Iod (1/2% *w*/*w*) at LED@385nm (a) under air and (b) in laminate and at LED@405nm, (c) under air and (d) in laminate; Figure S10: Evolution of the epoxy conversion of (1) EPOX and (2) hydrolysis extent versus irradiation time in the EPOX 75%/GPTMS 25% formulation in the presence of TX/Iod (1/2% *w*/*w*) at LED@385nm (a) under air and (b) in laminate, and at LED@405nm (c) under air and (d) in laminate; Figure S11: Evolution of the epoxy conversions of (1) DPDO, (2) GPTMS in the DPDO/GPTMS mixture and (3) hydrolysis extent vs. irradiation time of GPTMS 25 wt%/DPDO 75 wt% formulation in the presence of TX/Iod (1%/2% *w*/*w*) at LED@385 nm (a) under air and (b) in laminate, at LED@405 nm (c) under air and (d) in laminate, and (e) at LED@455 nm under air; Figure S12: ^1^H NMR spectrum of (2-Bromoethyl)thioxanthone; Figure S13: ^13^C NMR spectrum of (2-Bromoethyl)thioxanthone; Figure S14: FTIR spectrum of (2-Bromoethyl)thioxanthone; Figure S15: ^1^H NMR spectrum of (2-Ethylthioacetate)thioxanthone; Figure S16: ^13^C NMR spectrum of (2-Ethylthioacetate)thioxanthone; Figure S17: FTIR spectrum of (2-Ethylthioacetate)thioxanthone; Figure S18: ^1^H NMR spectrum of (2-Mercaptoethyl)thioxanthone; Figure S19: ^13^C NMR spectrum of (2-Mercaptoethyl)thioxanthone; Figure S20: IR spectrum of 2 (and 3)-(2-Mercaptoethyl)thioxanthone; Figure S21: ^1^H NMR spectrum of 2-(2-{[3-(trimethoxysilyl)propyl]sulfunyl}ethyl-9H-thioxanthen-9-one; Figure S22: ^13^C NMR spectrum of 2-(2-{[3-(trimethoxysilyl)propyl]sulfunyl}ethyl-9H-thioxanthen-9-one; Figure S23: IR spectrum of 2-(2-{[3-(trimethoxysilyl)propyl]sulfunyl}ethyl-9H-thioxanthen-9-one; Scheme S1: Reaction mechanism for the organic synthesis of 1b; Equation (S1): Rehm–Weller equation with E_ox_, E_red_, E_S_ (or E_T_), ∆Ec, and F are, respectively, the oxidation potential of the donor, the reduction potential of the acceptor, the excited singlet (or triplet) states energy of TXS, the Coulombic stabilization energy (negligible for most systems), and the Faraday constant.
