# Supplementary material for: Thioxanthone-Based Siloxane Photosensitizer for Cationic/Radical Photopolymerization and Photoinduced Sol–Gel Reactions
Source: Molecules. 2024 Jan 3;29(1):255. doi: 10.3390/molecules29010255 (PMC10780806; doi:10.3390/molecules29010255)
Supplement: Supplementary file 1 [file molecules-29-00255-s001.zip › molecules-2751771-supplementary.pdf]

SUPPORTING INFORMATION

Thioxanthone-Based Siloxane Photosensitizer for Cationic/Radical Photopolymerization and Photoinduced Sol-Gel Reactions

Thi-Thanh-Tam Nguyen [1], Louise Breloy [1], Agustin Rios De Anda [1], Hassan Hayek [1], Annalisa Chiappone [2], Jean-Pierre Malval [3], Daniel Grande [1] and Davy-Louis Versace [1,*]

[1] University Paris-Est Creteil, CNRS, ICMPE, UMR 7182, 94320 Thiais, France
[2] Dipartimento di Scienze Chimiche e Geologiche, Università degli Studi di Cagliari, Via Università 40, 09124 Cagliari, Italy
[3] Institut de Science des Matériaux de Mulhouse, UMR CNRS 7361, Université de Haute Alsace, 15 Rue Jean Starcky, 68057 Mulhouse, France
* Correspondence: davy-louis.versace@u-pec.fr

FIGURES

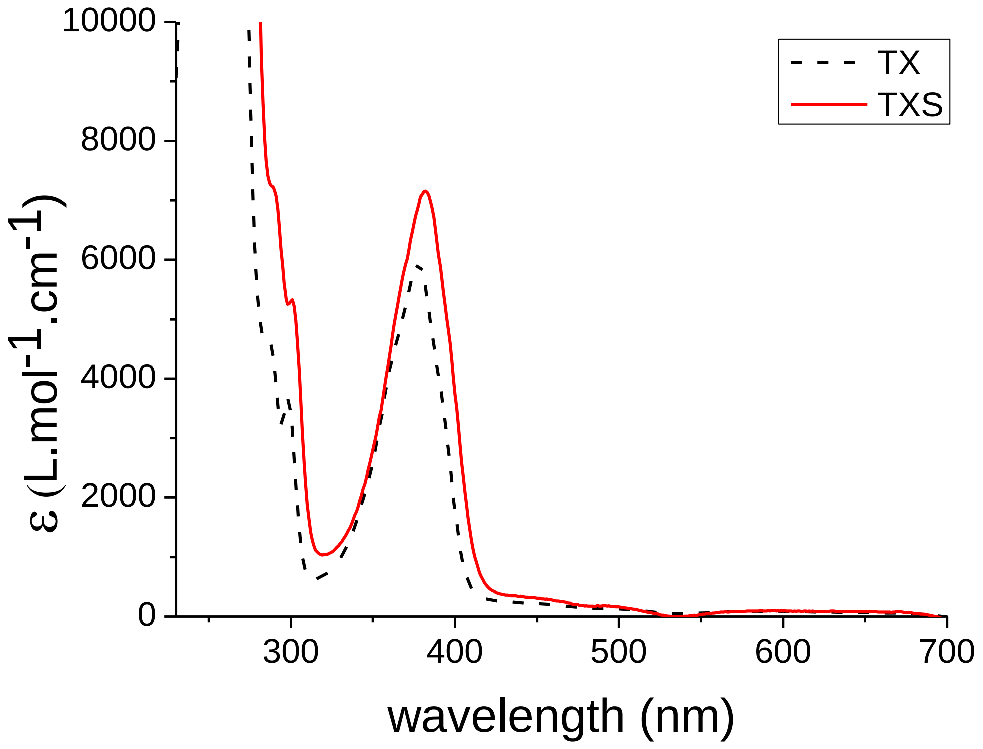

Figure S1. UV-Visible absorption spectra of TX and TXS in $CHCl_3$.

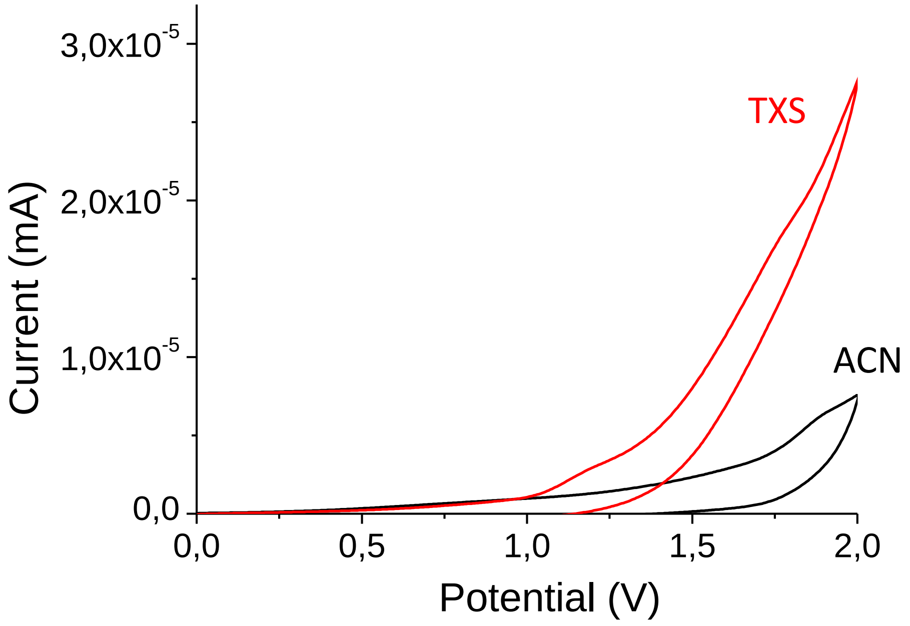

Figure S2. Cyclic voltammetry of TXS in ACN with tetrabutylammonium hexafluorophosphate ($[NEt_4PF_6]$ = 0.1 M in ACN) as supporting electrolyte. TXS solution was prepared at 10^{-3} M in this supporting electrolyte. Oxidation potential (E_{Ox}) was measured

between 0 and 2 V with a scan rate of 0.05 $V.s^{-1}$.

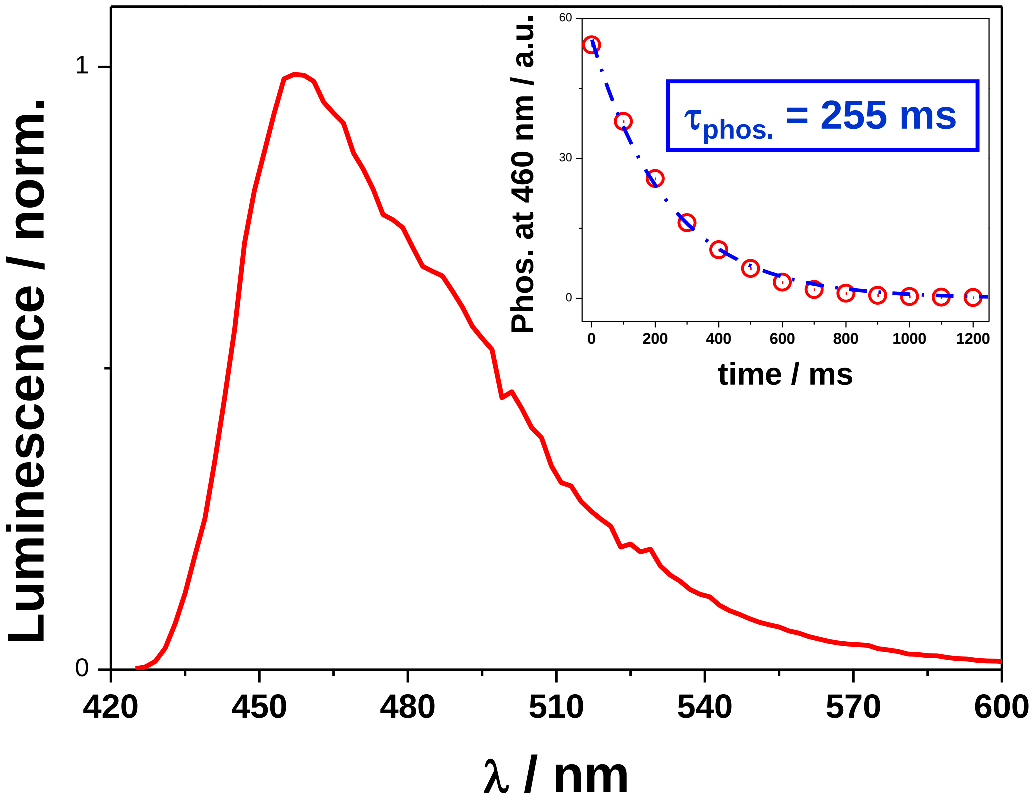

Figure S3. Normalized phosphorescence spectrum of **TXS** recorded in a glassy matrix of EtOH ($\lambda_{ex.}$ = 360 nm, delay = 2 ms, and time-gate = 25 ms). Inset: Time decay of the phosphorescence signal with the corresponding fitted curve.

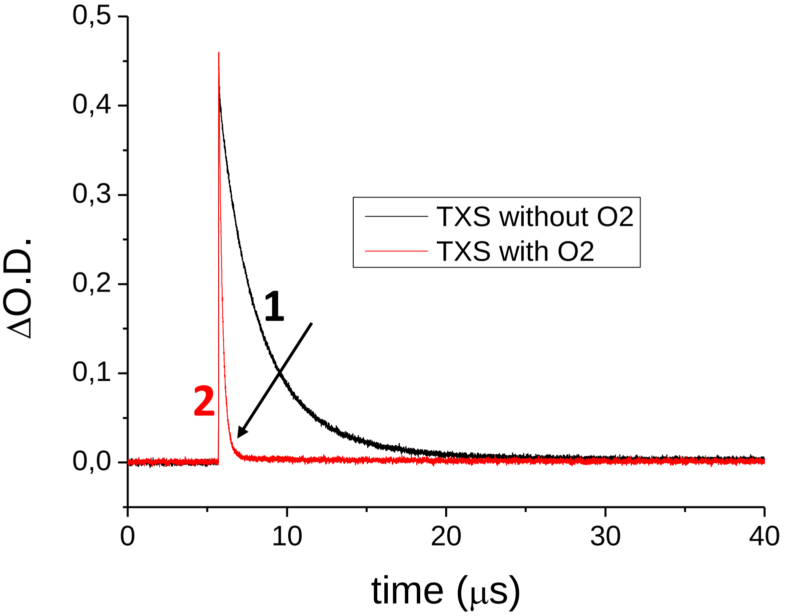

Figure S4. Decay of transient absorption signal at 640 nm after the light irradiation at 355 nm under argon atmosphere (black, curve 1) and under air (red, curve 2).

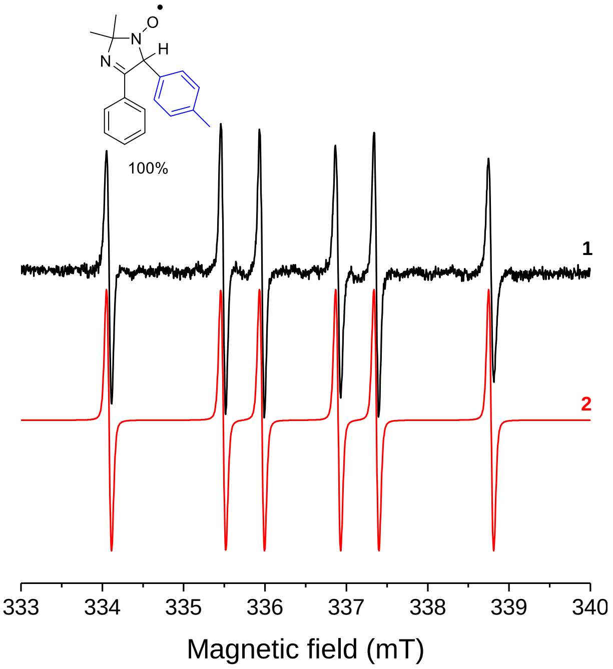

(a)

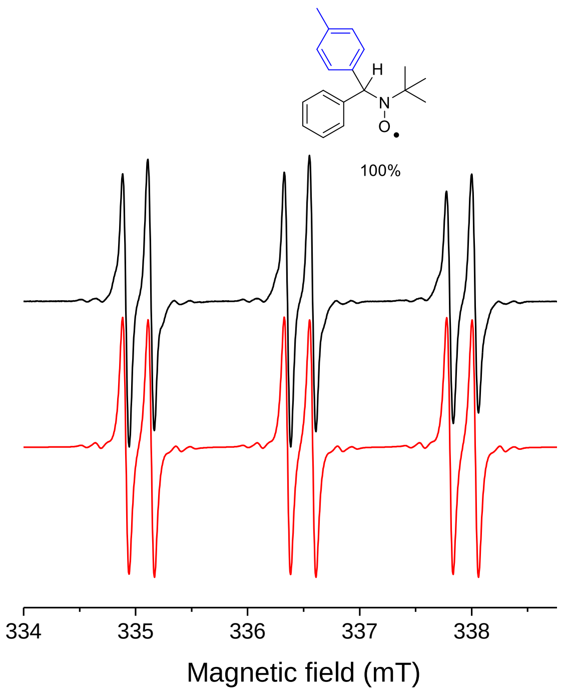

(b)

Figure S5. Exp erimental (1) and simulated (2) EPR spectra obtained during 450-s in situ exposure (LED@385 nm) of TXS/Iod in benzene solutions under argon in the presence of a) 2,2-Dimethyl-4-p henyl-2H-imidazole 1-oxide (DMPIO) and b) *N*-*tert*-Butyl-α-phenylnitrone (PBN) spin trapping agents. The (4-methyl)phenyl radical added to DMPIO molecule has the following spin-Hamiltonian parameters a_N = 1.412 mT, a_H= 1.888 mT, g = 2.0059; and the PBN-adduct with (4-methyl)phenyl radical is observed with the hyperfine coupling constants a_N = 1.451 mT, a_H= 0.227 mT, g = 2.0061. 100% of the signals corresponds to the DMPIO or PBN-adduct with (4-methyl)phenyl radical.

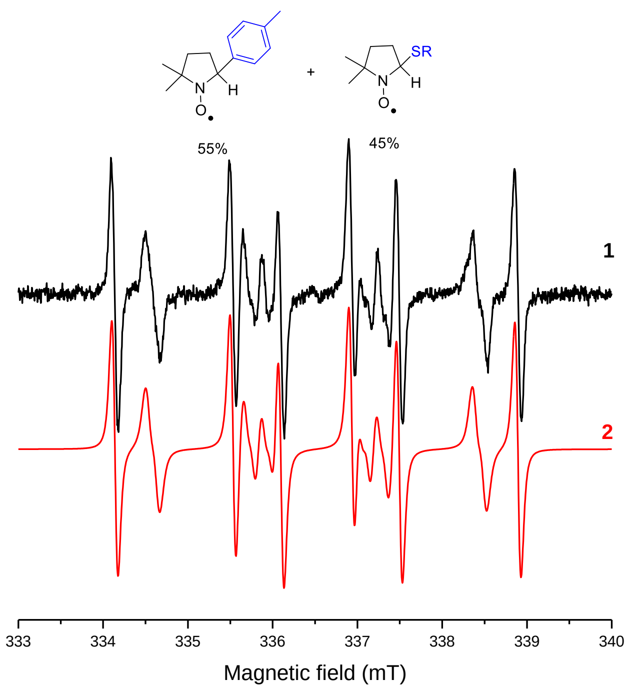

Figure S6. Experimental (1) and simulated (2) EPR spectra obtained during 450-s *in situ* exposure (LED@385 nm) of TXS/Iod in benzene solutions under argon in the presence of DMPO spin trapping agent. The irradiation of benzene solutions containing TXS/Iod/DMPO/benzene under inert atmosphere resulted in the immediate generation of characteristic six-line EPR signal of •DMPO-(4-methyl)phenyl adduct (55%) with the hyperfine coupling constants a_N = 1.401 mT, $a_{H\beta}$ = 1.967 mT; g = 2.0060, along with the superimposed signal assigned to the DMPO-adduct with sulfur-centered radical (•DMPO-SR, 45%) with the hyperfine coupling constants a_N = 1.359 mT, $a_{H\beta}$ = 1.149 mT, $a_{H\gamma}$ = 0.084 mT and $a_{H\gamma}$ = 0.025 mT.

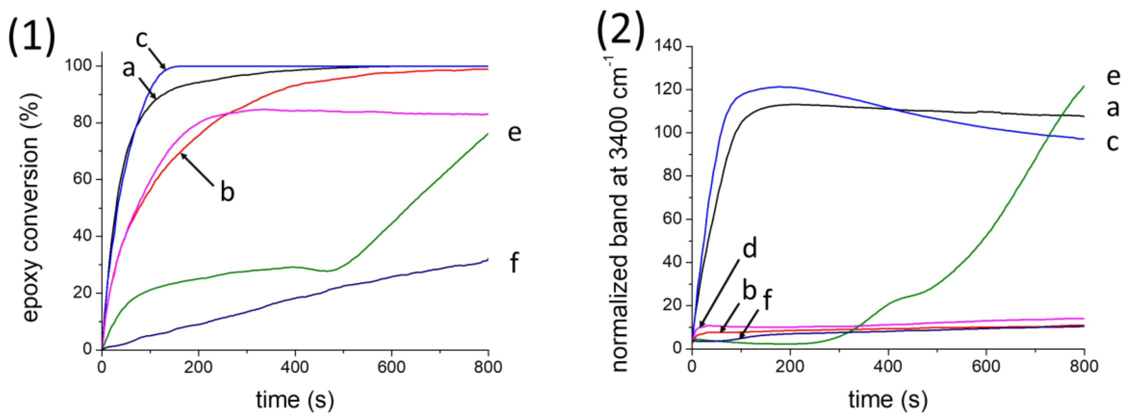

Figure S7. Evolution of the epoxy conversion of (1) GPTMS and (2) hydrolysis extent versus irradiation time in the presence of **TX/Iod** (1/2% w/w) upon LED@385nm (a) under air and (b) in laminate, upon LED@405nm (c) under air and (d) in laminate, and upon LED@455nm (e) under air and (f) in laminate

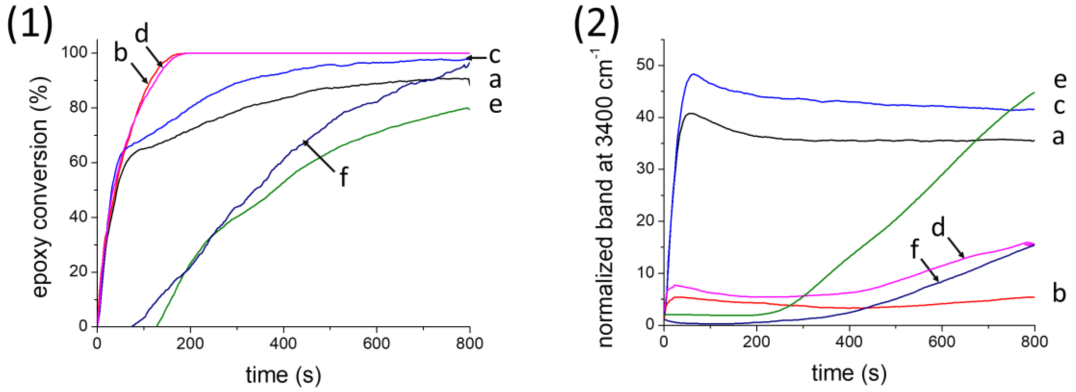

Figure S8. Evolution of the epoxy conversion of (1) GPTMS and (2) hydrolysis extent versus irradiation time in the presence of **TXS/Iod** (1/2% w/w) upon LED@385nm (a) under air and (b) in laminate, upon LED@405nm (c) under air and (d) in laminate, and upon LED@455nm (e) under air and (f) in laminate

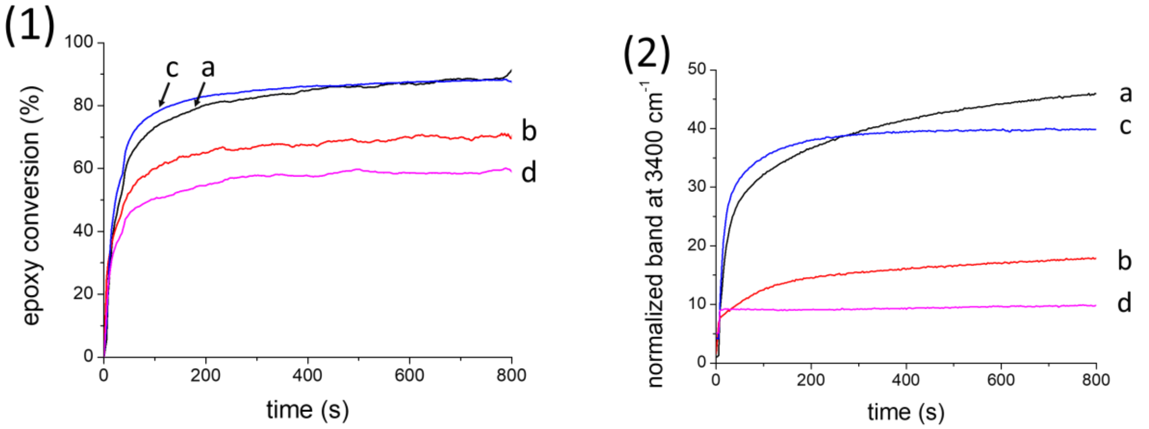

Figure S9. Evolution of the epoxy conversion of (1) EPOX and (2) hydrolysis extent versus irradiation time in the EPOX 75%/GPTMS 25% formulation in the presence of **TXS/Iod** (1/2% w/w) upon LED@385nm (a) under air and (b) in laminate, upon LED@405nm (c) under air and (d) in laminate

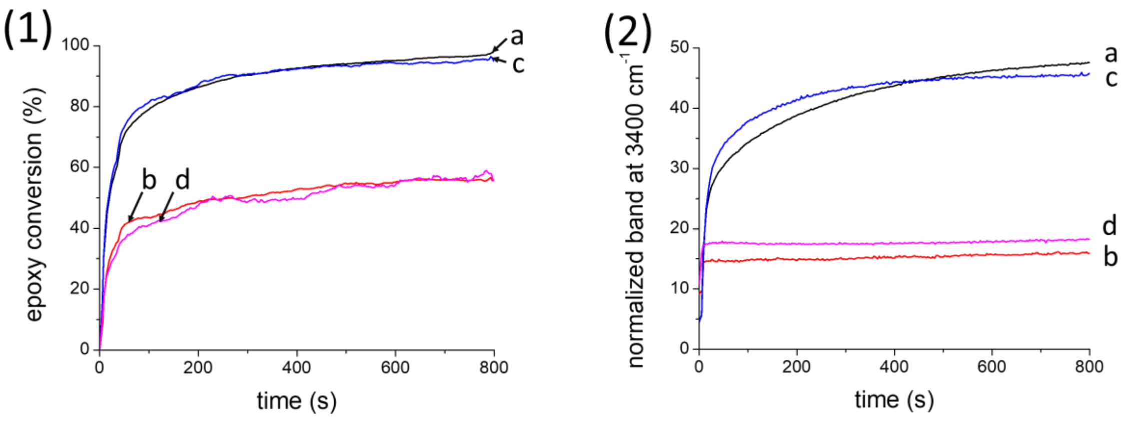

Figure S10. Evolution of the epoxy conversion of (1) EPOX and (2) hydrolysis extent versus irradiation time in the EPOX 75%/GPTMS 25% formulation in the presence of **TX/Iod** (1/2%

w/w) upon LED@385nm (a) under air and (b) in laminate, upon LED@405nm (c) under air and (d) in laminate.

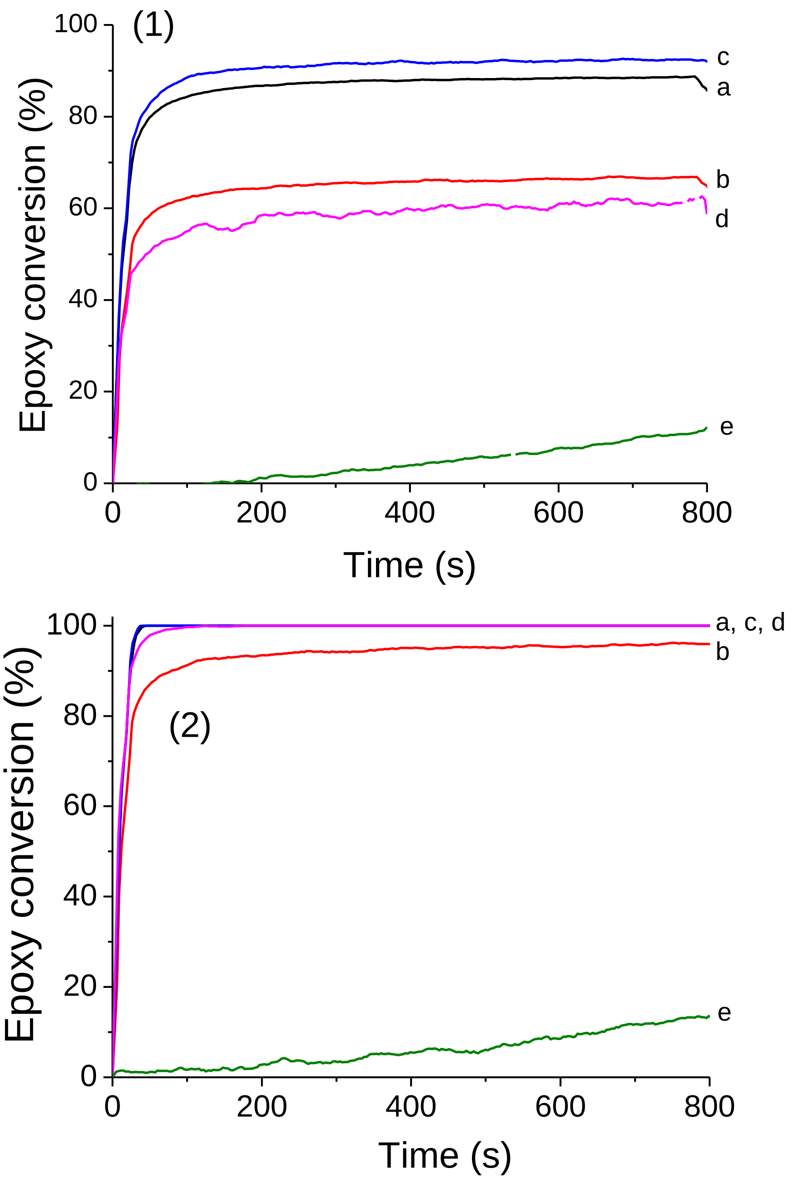

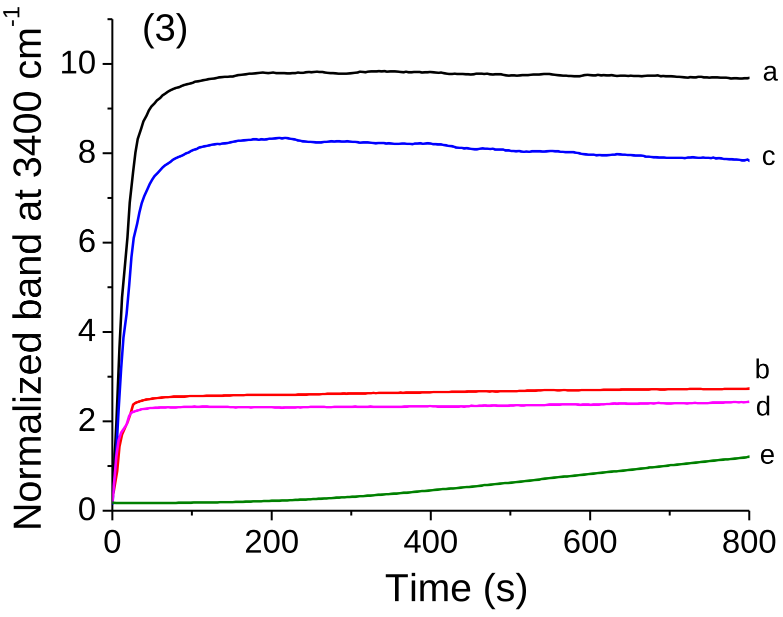

Figure S11. Evolution of the epoxy conversions of (1) DPDO, (2) GPTMS in the DPDO/GPTMS mixture and (3) hydrolysis extent vs irradiation time of GPTMS 25 wt% / DPDO 75 wt% formulation in the presence of **TX/Iod** (1%/2% w/w) upon LED@385 nm (a) under air and (b) in laminate, upon LED@405 nm (c) under air and (d) in laminate, and (e) upon LED@455 nm under air.

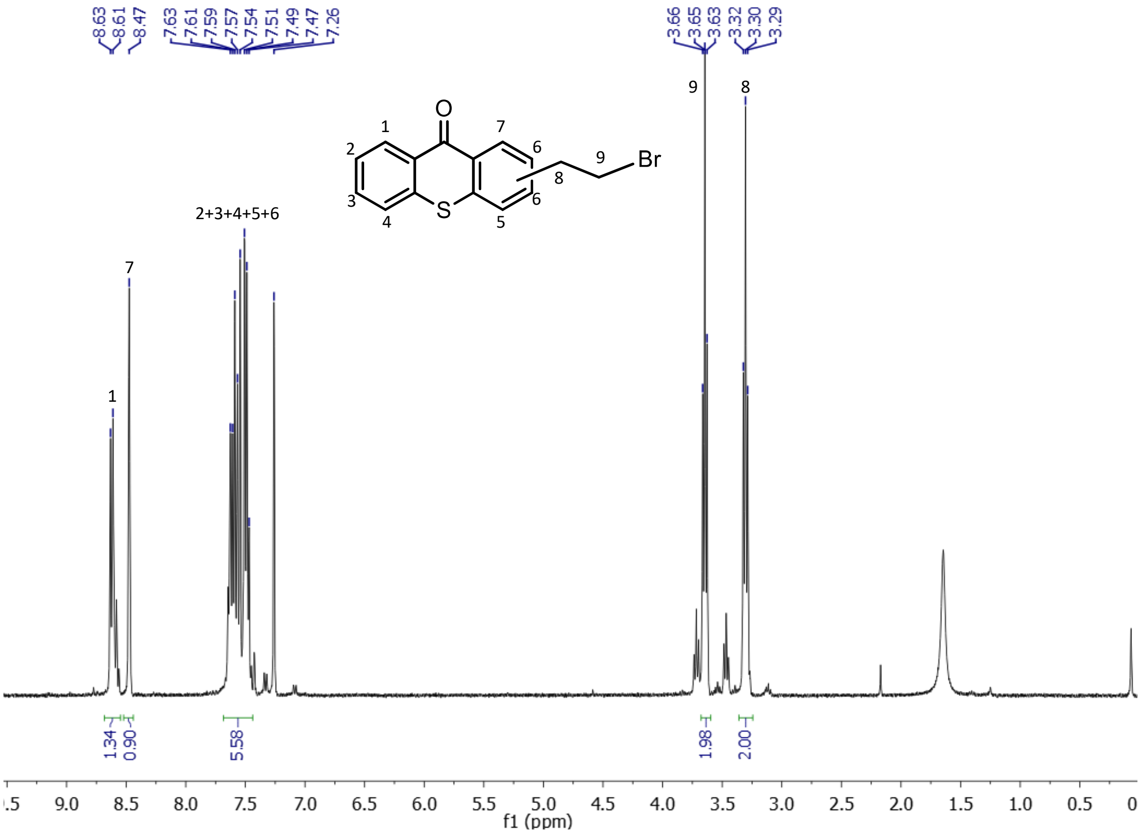

Figure S12. ^{1}H NMR spectrum of (2-Bromoethyl)thioxanthone

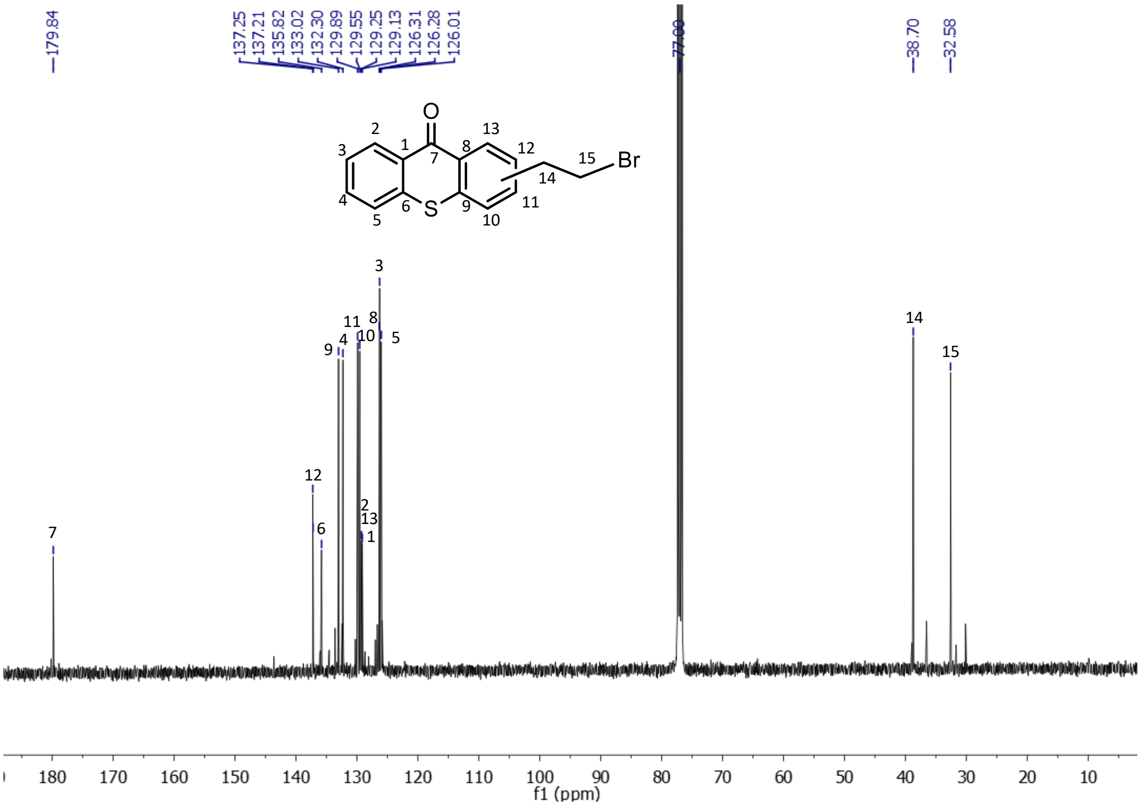

Figure S13. ^{13}C NMR spectrum of (2-Bromoethyl)thioxanthone

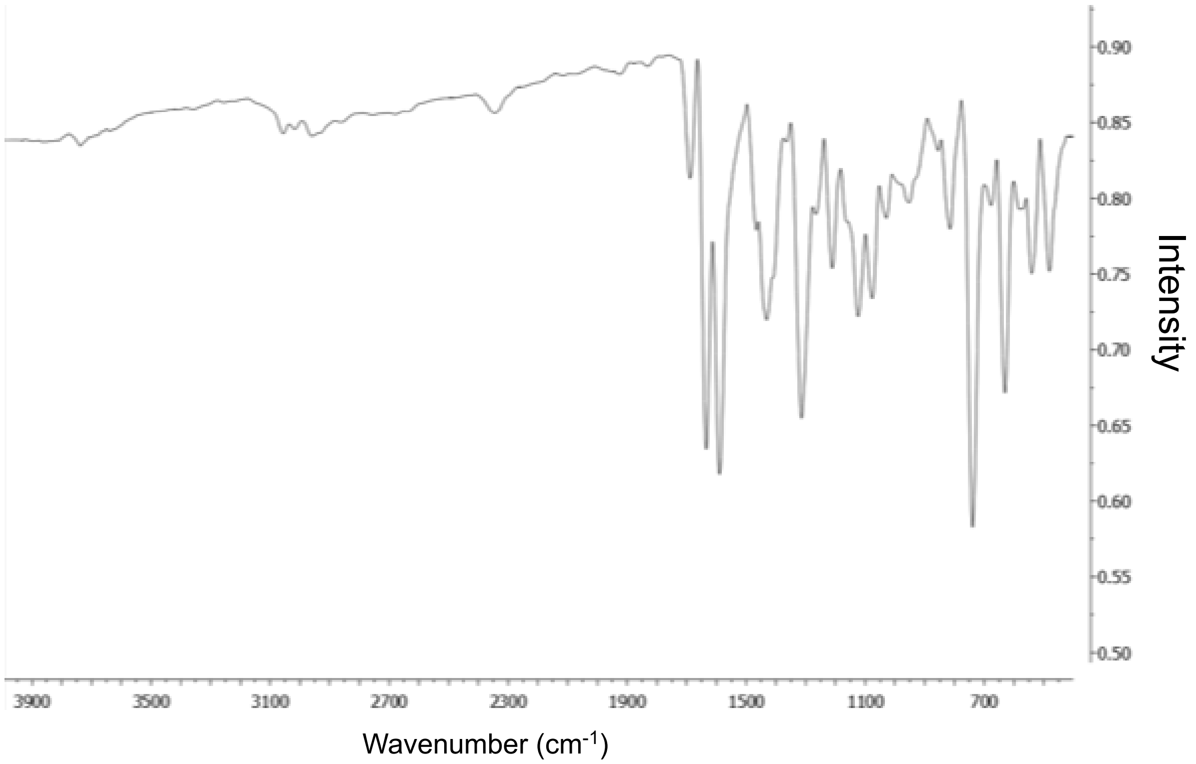

Figure S14. FTIR spectrum of (2-Bromoethyl)thioxanthone

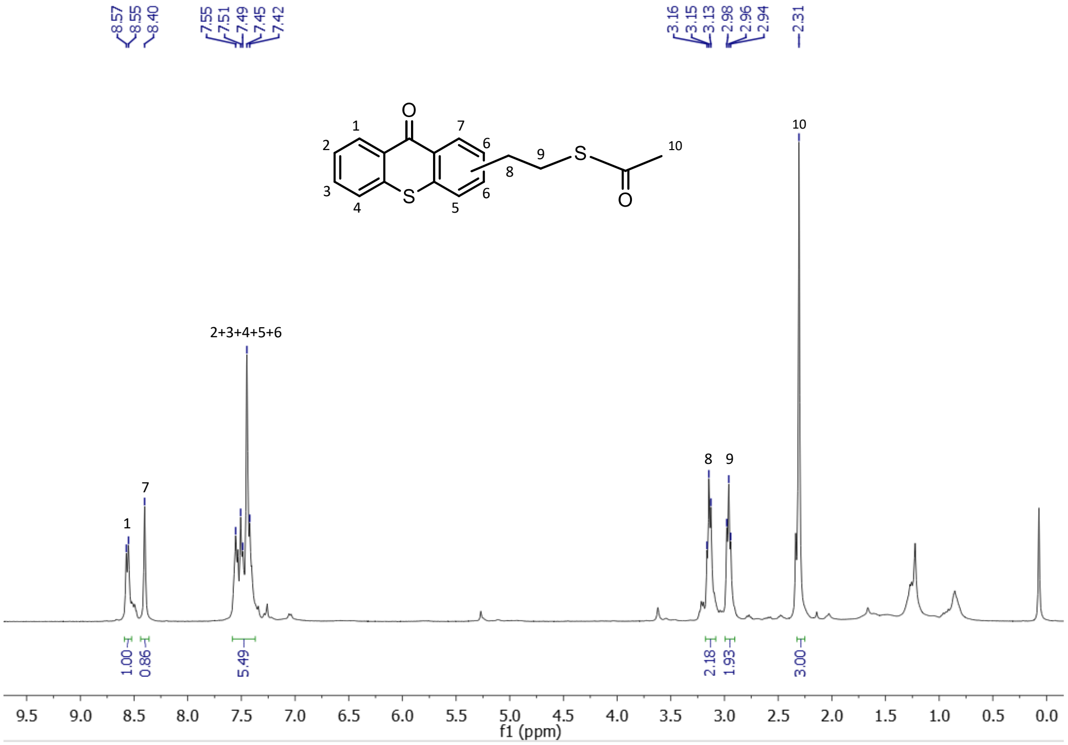

Figure S15. ^{1}H NMR spectrum of (2-Ethylthioacetate)thioxanthone

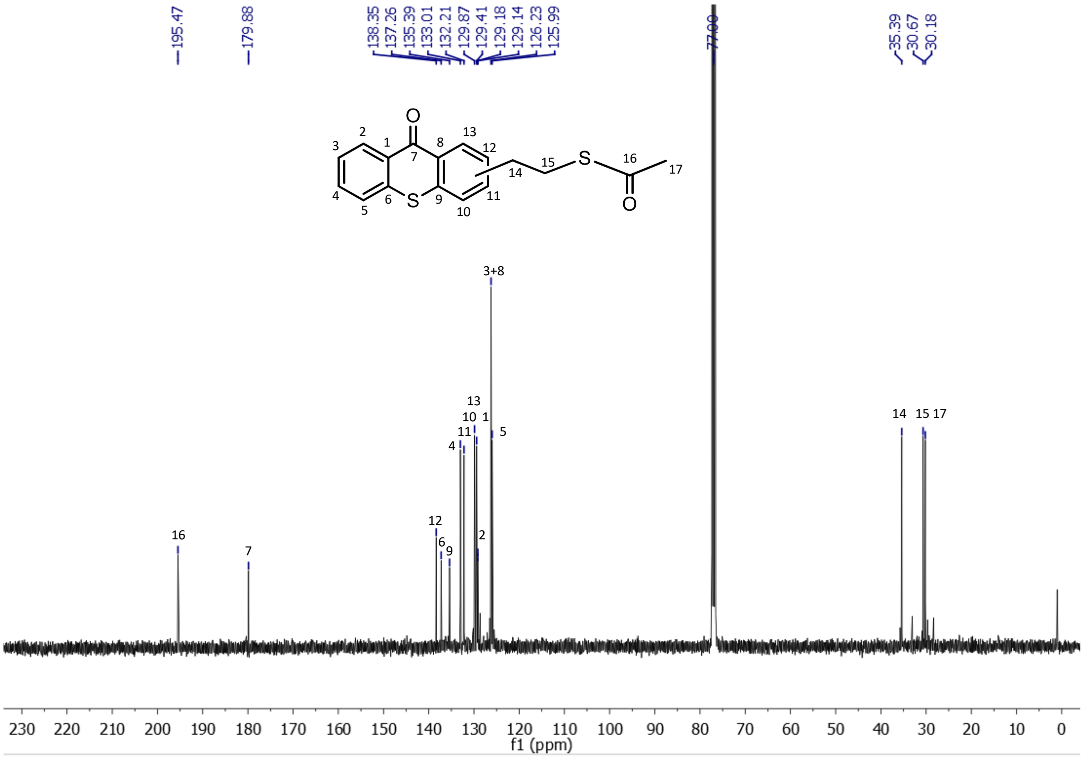

Figure S16. ^{13}C NMR spectrum of (2-Ethylthioacetate)thioxanthone.

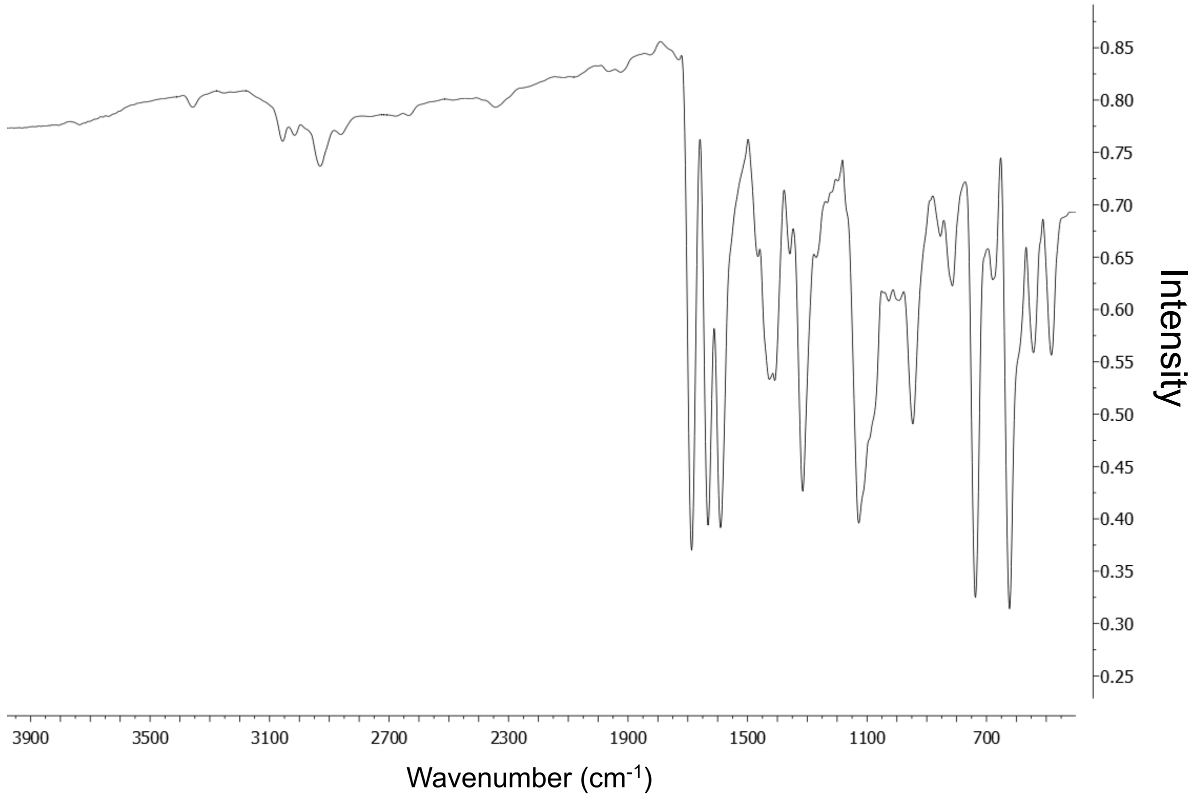

Figure S17. FTIR spectrum of (2-Ethylthioacetate)thioxanthone.

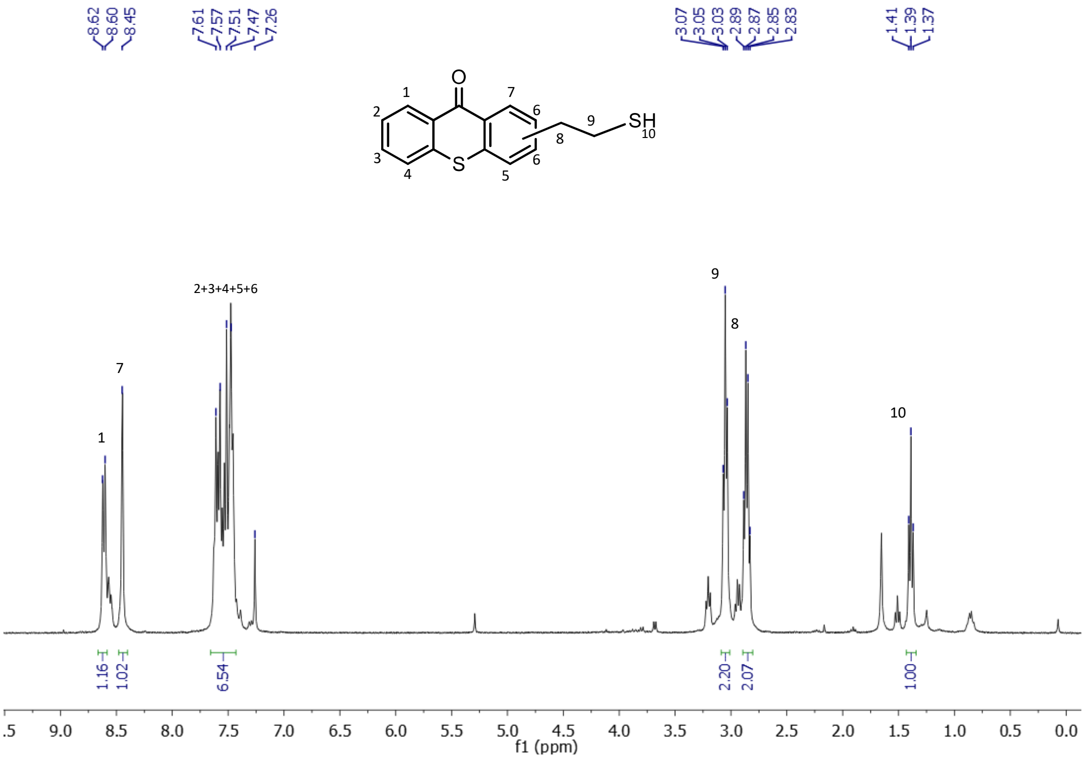

Figure S18. ^{1}H NMR spectrum of (2-Mercaptoethyl)thioxanthone.

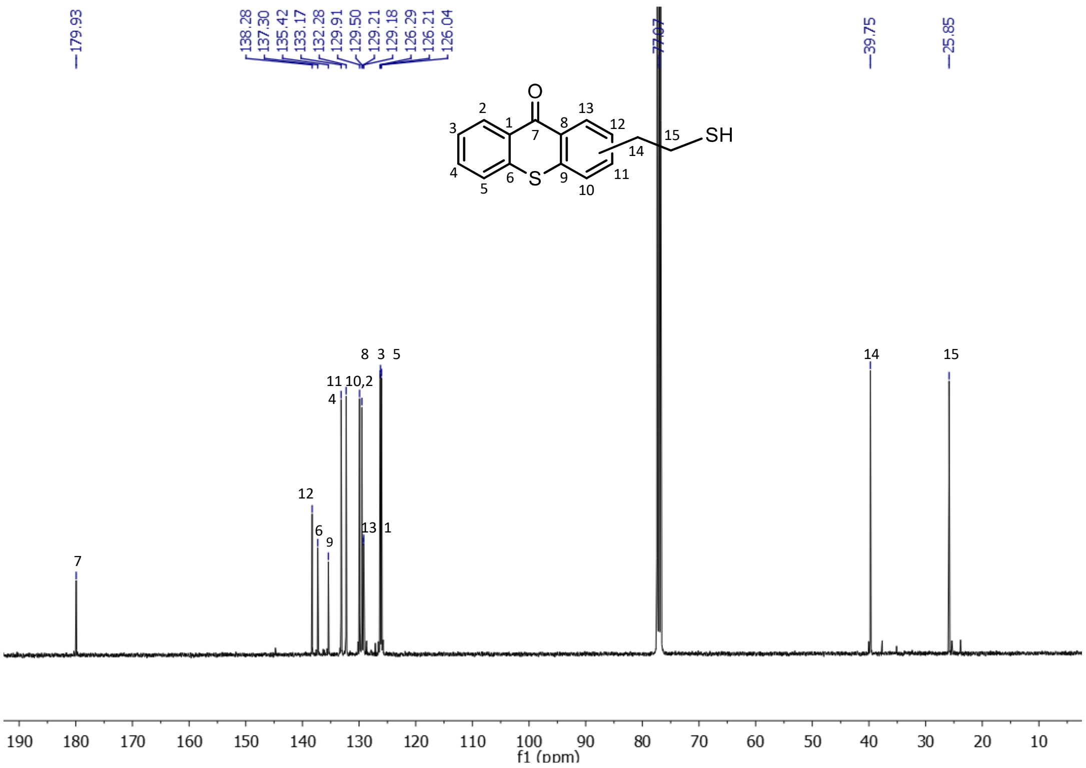

Figure S19. ^{13}C NMR spectrum of (2-Mercaptoethyl)thioxanthone

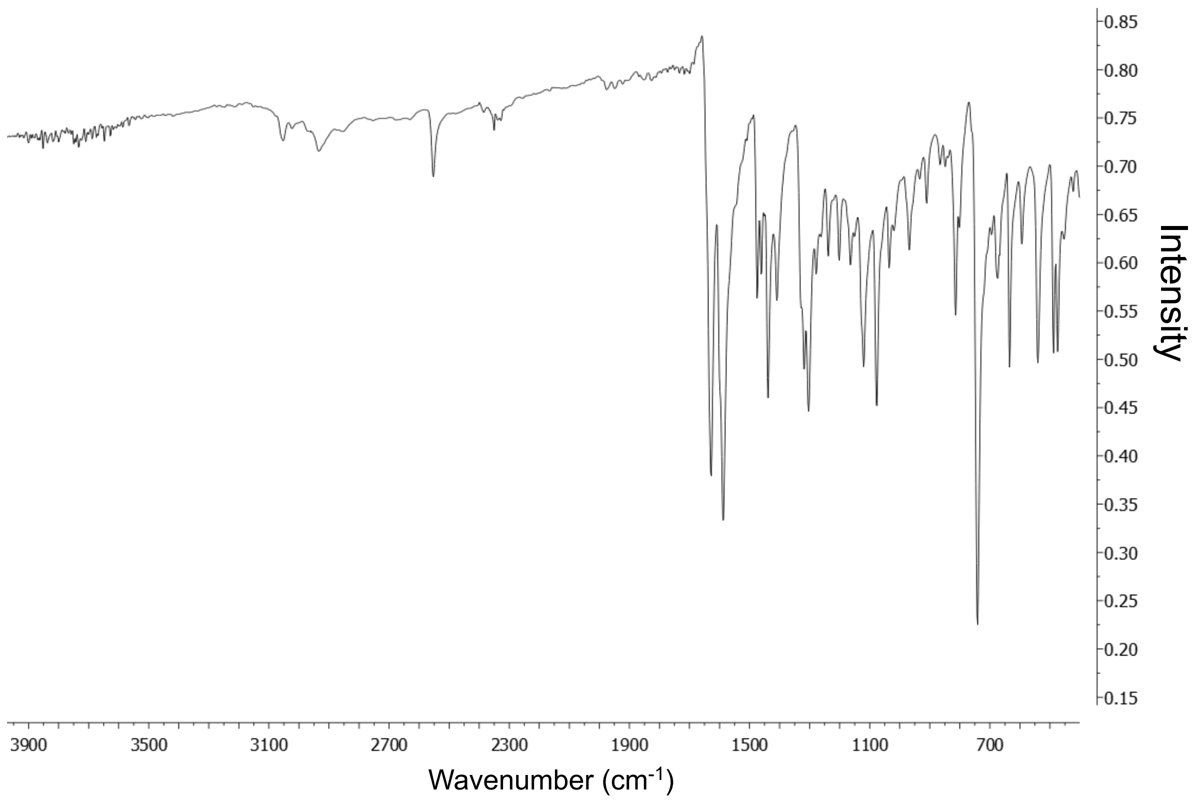

Figure S20. IR spectrum of 2 (and 3)-(2-Mercaptoethyl)thioxanthone

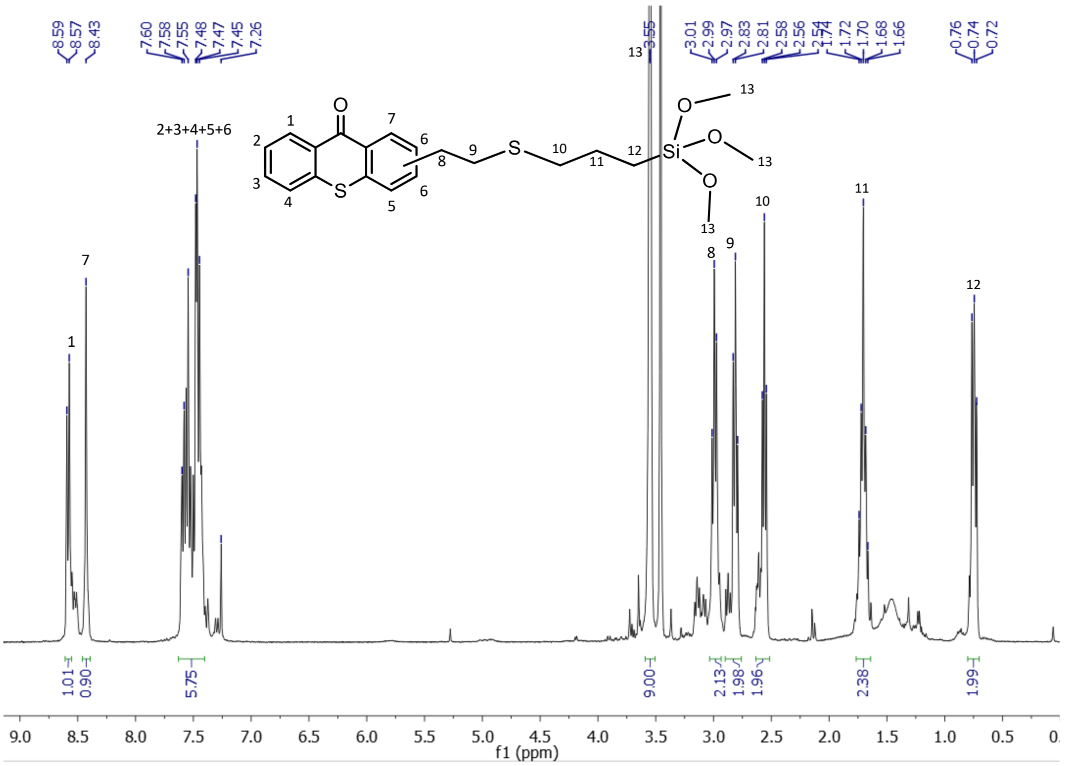

Figure S21. ^{1}H NMR spectrum of 2-(2-{[3-(trimethoxysilyl)propyl]sulfunyl}ethyl-9H-thioxanthen-9-one (TXS).

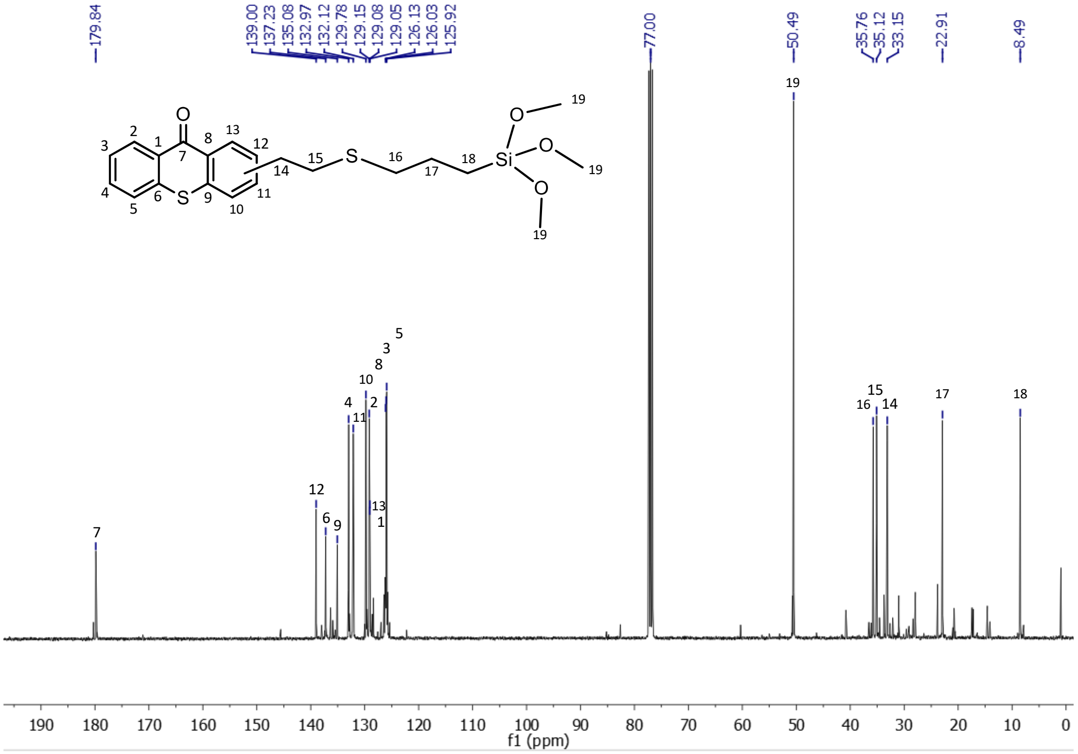

Figure S22. ^{13}C NMR spectrum of 2-(2-{[3-(trimethoxysilyl)propyl]sulfunyl}ethyl-9H-thioxanthen-9-one (TXS).

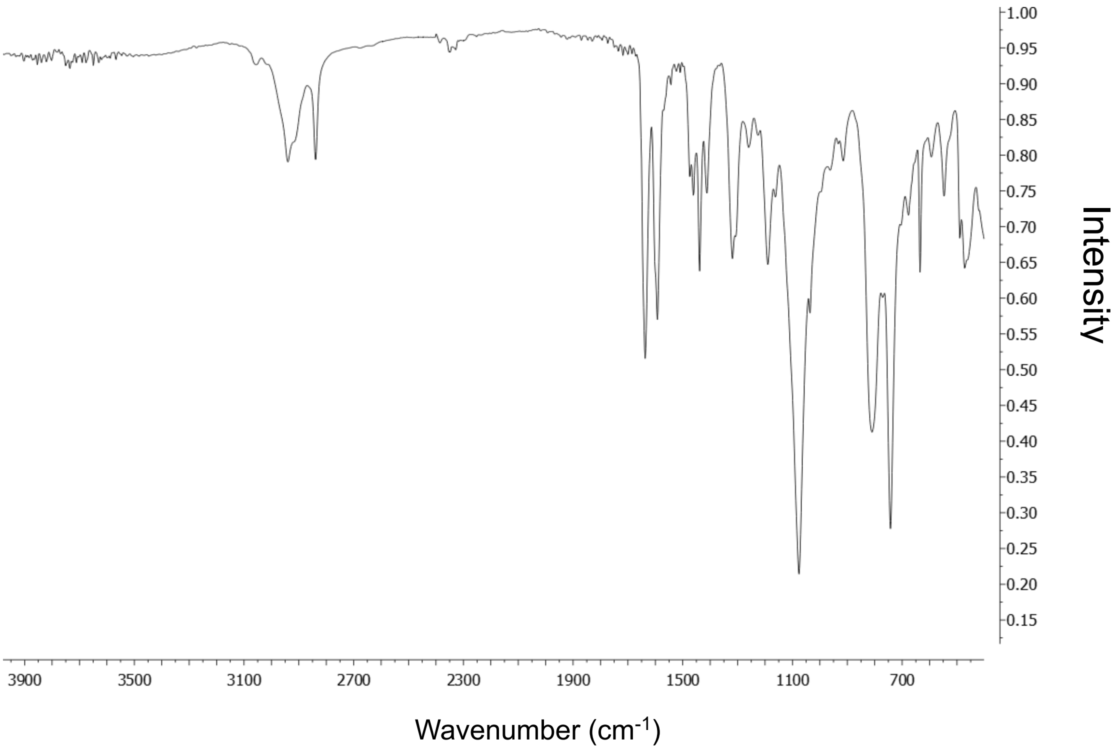

Figure S23. IR spectrum of 2-(2-{[3-(trimethoxysilyl)propyl]sulfunyl}ethyl-9H-thioxanthen-9-one (TXS).

SCHEME

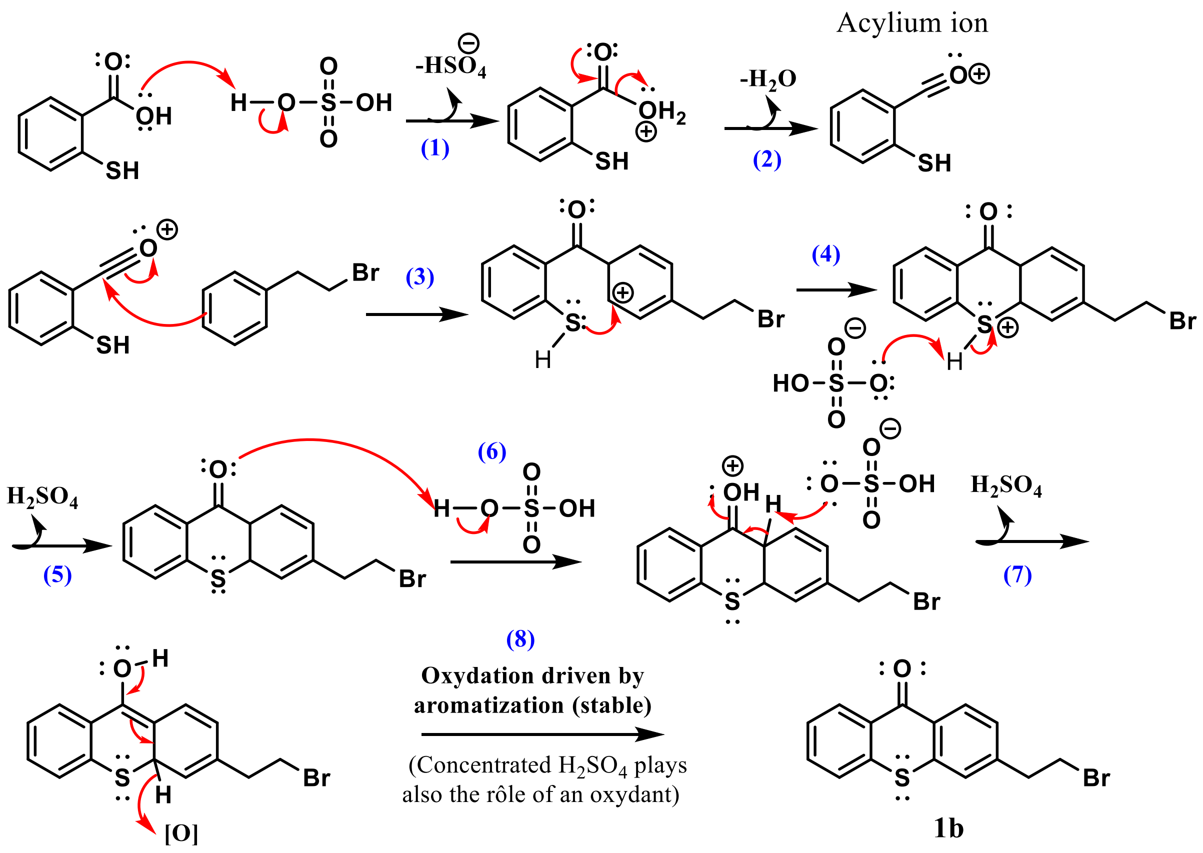

Scheme S1. Reaction mechanism for the organic synthesis of **1b**

EQUATION

$$\Delta G_{eT} = F \times (E_{ox}(\text{Donor}) - E_{red}(\text{Acceptor})) - E_S \text{ (or } E_T) + \Delta E_c$$

Equation S1. Rehm-Weller equation with E_{ox}, E_{red}, E_S (or E_T), ΔE_c and F are respectively the oxidation potential of the donor, the reduction potential of the acceptor, the excited singlet (or triplet) states energy of TXS, the Coulombic stabilization energy (negligible for most systems) and Faraday constant.
